# Programmed Fabrication of Vesicle‐Based Prototissue Fibers with Modular Functionalities

**DOI:** 10.1002/advs.202409066

**Published:** 2025-02-10

**Authors:** Tomoya Kojima, Kouichi Asakura, Pierangelo Gobbo, Taisuke Banno

**Affiliations:** ^1^ Department of Applied Chemistry Keio University 3‐14‐1 Hiyoshi, Kohoku‐ku Yokohama Kanagawa 223‐8522 Japan; ^2^ Department of Chemical and Pharmaceutical Sciences University of Trieste Via L. Giorgieri 1 Trieste 34127 Italy; ^3^ National Interuniversity Consortium of Materials Science and Technology Unit of Trieste Via G. Giusti 9 Firenze 50121 Italy

**Keywords:** bioinspired material, protocell, prototissue, soft fiber, vesicle

## Abstract

Multicellular organisms have hierarchical structures where multiple cells collectively form tissues with complex 3D architectures and exhibit higher‐order functions. Inspired by this, to date, multiple protocell models have been assembled to form tissue‐like structures termed prototissues. Despite recent advances in this research area, the programmed assembly of protocells into prototissue fibers with emergent functions still represents a significant challenge. The possibility of assembling prototissue fibers will open up a way to a novel type of prototissue subunit capable of hierarchical assembly into unprecedented soft functional materials with tunable architectures, modular and distributed functionalities. Herein, the first method to fabricate freestanding vesicle‐based prototissue fibers with controlled lengths and diameters is devised. Importantly, it is also shown that the fibers can be composed of different specialized modules that, for example, can endow the fiber with magnetotaxis capabilities, or that can work synergistically to take an input diffusible chemical signals and transduce it into a readable fluorescent output through a hosted enzyme cascade reaction. Overall, this research addresses an important challenge of prototissue engineering and will find important applications in 3D bio‐printing, tissue engineering, and soft robotics as next‐generation bioinspired materials.

## Graphical abstract

The fabrication of freestanding vesicle‐based prototissue fibers is reported. The lengths, diameters, and shapes of the fibers can be well‐controlled. Multimodular fibers with different prototissues can be fabricated. Focusing on modularity, manipulation functionalities are obtained when connected with magnetic fibers. Connection of input and output fiber modules results in spatiotemporal directional signal transduction.

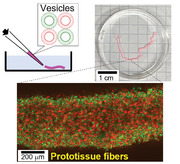

## 1 Introduction

Multicellular organisms have hierarchical structures where multiple cells assemble into living tissues to form complex 3D architectures.^[^ ^1^ ^]^ Living tissues exhibit higher‐order behaviors (e.g., signal transduction, contractility, phototropism, etc.), meaning that the cell units are able to interact with one another and generate a novel property of the ensemble, which is on a higher level.^[^ ^2^ ^]^ Creating such hierarchical structures of living tissues artificially can be one of the most significant breakthroughs in bioinspired material science.^[^ ^3^ ^]^ This could lead to the development of a next generation of soft materials for diverse applications ranging from soft robotics to microbioreactor technology and tissue engineering.^[^ ^4^ ^]^ Moreover, modularity is one of the fundamental aspects of biological organization where different building blocks cooperate to form complex biological systems.^[^ ^5^ ^]^ In terms of modularity, multicellular organisms are considered to be composed of multiple specialized modules such as organs, tissues, and cells that are fully integrated and continuously interact to provide higher‐order functions. Thus, the development of tissue‐like structures comprising different specialized modules could lead to the integration of other building blocks into one hierarchical structure with synergistic functionalities.

In recent years, research efforts in this direction have led to the concept of protocells in the field of bottom‐up synthetic biology where cell functions can emerge from inanimate molecules and materials.^[^ ^6^ ^]^ Protocells are synthetic microcompartmentalized systems such as vesicles,^[^ ^7^ ^]^ coacervates,^[^ ^8^ ^]^ DNA droplets,^[^ ^9^ ^]^ lipid‐coated aqueous droplets having droplet interface bilayers (DIB),^[^ ^10^ ^]^ and proteinosomes,^[^ ^11^ ^]^ which are chemically programmed to mimic at least one fundamental aspect of a living cell. Based on the concept of the protocells, prototissues have been created as 3D assemblies of multiple protocells and can be designed and chemically programmed to mimic basic aspects of living tissues, such as chemo‐mechanical transduction,^[^ ^12^ ^]^ signal transduction,^[^ ^13^ ^]^ conversion of external signals into changes in phenotype properties,^[^ ^14^ ^]^ and enhanced survivability against predators.^[^ ^15^ ^]^

One of the most significant challenges of prototissue engineering is the fabrication of robust freestanding prototissues from the direct adhesion of protocells units. In this regard, the Gobbo group has succeeded in developing a programmed assembly of proteinosomes. Micrometer‐sized prototissue spheroids comprising proteinosomes were first reported using the Pickering emulsion procedure via an interfacial strain‐promoted alkyne‐azide cycloaddition (I‐SPAAC).^[^ ^12^ ^]^ Later, they devised a floating mold technique to make protocellular materials where millimeter‐sized proteinosome‐based prototissues can be made in any shape.^[^ ^16^ ^]^ Another example of technique that allowed for the assembly of prototissues with desired shapes is the aqueous droplets 3D‐printing developed by Bayley and co‐workers.^[^ ^17^ ^]^

Among the many protocell models, vesicles are the most biomimetic protocells because, similarly to the membranes of biological cells, their membrane is composed of a lipid bilayer.^[^ ^7^ ^]^ Thus, the controlled assembly of multiple vesicles could lead to important advancements toward the bottom‐up chemical construction of fully functioning forms of prototissues. Some methods to promote the aggregation of multiple vesicles using light,^[^ ^18^ ^]^ metal ions,^[^ ^19^ ^]^ cadherins,^[^ ^20^ ^]^ and squeezed sponges,^[^ ^21^ ^]^ or by the formation of streptavidin–biotin pair,^[^ ^22^ ^]^ lectin–glycan pair,^[^ ^23^ ^]^ and DNA complementary strands^[^ ^24^ ^]^ have been reported in the literature. However, these methods could not provide freestanding prototissues with controlled shapes. Recent research reported programmable capillary‐induced assemblies of vesicles, which however had to be immersed in oil with consequent limited technological applications.^[^ ^25^ ^]^ Optical tweezers have also been used to construct vesicle networks,^[^ ^26^ ^]^ but with this technique it is technically challenging to achieve millimeter‐sized assemblies. External fields such as magnetic fields^[^ ^27^ ^]^ and acoustic fields^[^ ^28^ ^]^ have been applied as well to assemble vesicles, which resulted in the structure dissipation when the external field was turned off. Another approach to the bottom‐up chemical construction of prototissues relies on the embedment of protocells within a hydrogel matrix.^[^ ^29^ ^]^ However, this method does not allow for direct protocell–protocell adhesions. To date, the fabrication of robust, large (millimeter to centimeter size) and freestanding vesicle‐based prototissues remains a considerable challenge. The programmed assemblies of multiple vesicles into prototissues with controlled shapes will pave the way toward the application of prototissues not only in the field of bioengineering, regenerative medicine and tissue engineering, but also in soft robotics, filtration technologies, and photocatalysis. Recently, our group reported the possibility of using salt bridges to assemble vesicle consortia with higher‐order cooperative functionalities; however, their controlled assembly into freestanding prototissues with controlled shapes was not achieved.^[^ ^30^ ^]^

To complete this challenging scenario, the possibility of producing prototissues in the form of freestanding fibers of directly interconnected vesicles with controlled lengths, diameters, and shapes still remains unexplored. The possibility of producing prototissue fibers not only will lead to an unprecedented type of prototissue subunit capable of hierarchical assembly into soft functional materials with tunable architectures, modular and distributed functionalities, but they could also open up a way to the 3D printing of much more complex and larger prototissue architectures. Herein, we describe the first method to fabricate robust freestanding millimeter‐sized modular prototissue fibers with controlled lengths and diameters. Importantly, we showed that modular prototissue fibers with distributed functionalities could be assembled from the controlled adhesion of fiber subunits composed of specialized protocells. We demonstrated this by assembling prototissue fibers composed of a module for magnetotaxis capable of dragging the rest of the fiber, or comprising modules capable of working synergistically to take an input diffusible chemical signal and transduce it into a readable fluorescent output through a hosted enzyme cascade reaction. From a general perspective, our results address an important challenge of prototissue engineering and will find important applications in soft robotics, microbioreactor technologies, and flow chemistry.

## 2 Results and Discussion

### 2.1 Fabrication of Vesicle‐Based Prototissue Fibers

**Figure 1:**
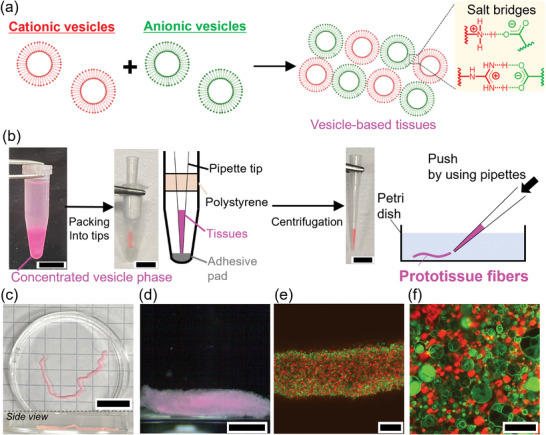
Fabrication of prototissue fibers. a) Scheme describing the formation of vesicle–vesicle adhesions via salt bridges by mixing cationic and anionic vesicles in 1:1 ratio. b) Scheme describing our method to assemble prototissue fibers supported by photos of the different steps involved in the experimental procedure. Scale bar: 1 cm. c) Photo and d) digital microscopy images of a prototissue fiber immersed in HEPES buffer (10 × 10^−3^ m, pH 7.2). Scale bar: 1 cm. e,f) Two‐photon excitation microscopic images of the prototissue fiber in (c) and (d). Texas Red (red fluorescence) and NBD (green fluorescence) were used to tag the cationic and anionic vesicles, respectively. Scale bar: (e) 200 µm, (f) 50 µm.

Based on thin‐film hydration methods, cationic vesicles were prepared by mixing 1‐palmitoyl‐2‐oleoyl‐*sn*‐glycero‐3‐phosphocholine (POPC, 2 × 10^−3^ m) and 4 mol% amphiphilic amines or guanidium chloride (80 × 10^−6^ m) in HEPES buffer (10 × 10^−3^ m, pH 7.2). Likewise, anionic vesicles were prepared by mixing POPC (2 × 10^−3^ m) and 4 mol% amphiphilic carboxylic acids (80 × 10^−6^ m) in HEPES buffer (10 × 10^−3^ m, pH 7.2). POPC was chosen as the major components of lipids because POPC membranes have moderate membrane fluidities which both enhances the attachment of different vesicles and suppresses their fusion. Size distribution and *ζ*‐potential of the vesicles were characterized (Figures S1 and S2, Supporting Information). The obtained dispersions of cationic and anionic vesicles were mixed at a ratio of 1:1 (**Figure** **1**a and S3, Supporting Information). Salt bridge‐mediated adhesion of vesicles was used to make assemblies of vesicles.^[^ ^30^ ^]^ Salt bridges are intermolecular interactions comprising ionic and hydrogen bonds, which selectively interact between ammonium/guanidium and carboxylate ions. IR measurement clearly showed the intermolecular interactions (Figure S4, Supporting Information). The mixed 1:1 dispersion of cationic and anionic vesicles was centrifuged at 16 000 *g* for 10 min to obtain a concentrated vesicle phase. The concentrated vesicle phase was loaded into a pipette tip, and the tip was set to a device made of a microcentrifuge tube, a polystyrene pipette tip holder, and an adhesive pad used to plug the pipette tip (Figure 1b). The device enabled us to load and pack the vesicles inside the pipette tip using mild centrifugation (400 *g* for 5 min). The pipette tip was then carefully removed from the microcentrifuge tube, attached to a mechanical pipette, and the packed vesicles were extruded in HEPES buffer (pH 7.2) to obtain a stable and freestanding prototissue fiber (Figure 1c,d, Figure S5 and Video S1, Supporting Information). The details of the prototissue fiber were captured using a two‐photon excitation fluorescence microscope. Figure 1e,f shows that the fiber was composed of Texas Red‐tagged (red fluorescence) cationic and NBD‐tagged (green fluorescence) anionic vesicles which adhered to one another via salt bridge interactions, and that the vesicles retained their structures even after the centrifugation processes. Prototissue fibers were found to be robust as they retained their structures over one week (Figure S6, Supporting Information). Importantly, control experiments carried out using only cationic or anionic vesicles to try to assemble the prototissue fibers showed prompt dispersion of the packed vesicles extruded from the tip, indicating that salt bridges are key to achieve vesicle‐vesicle adhesions and their fiber assembly (Figure S7, Supporting Information).

**Figure 2:**
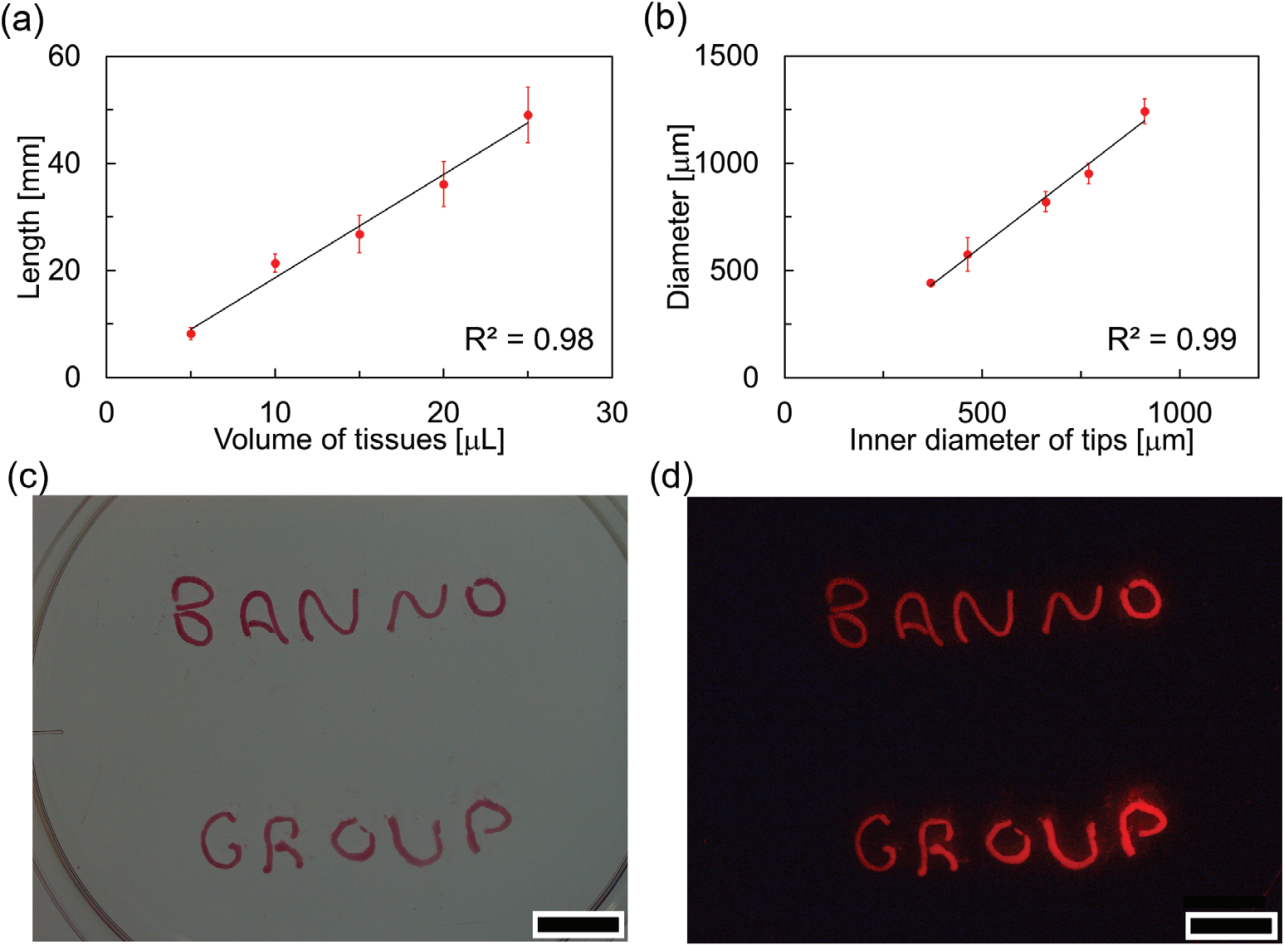
Controllability of prototissue fiber lengths and diameters. a) Control of the length of prototissue fibers when changing the volume of the concentrated vesicle phase. b) Control of the diameter of prototissue fibers when changing inner diameter of the used pipette tips. c) Bright‐field and d) fluorescent images of our group's logo “BANNO GROUP” made of prototissue fibers containing Nile red as fluorescent dyes. *λ*_ex_ = 530 nm, *λ*_em_ > 570 nm. Scale bar: 1 cm.

Subsequently, we explored the versatility of our new method for the generation of prototissue fibers, and showed that it can be used to produce fibers of different lengths and diameters. To control the length of the prototissue fibers, the volume of the concentrated vesicle phase loaded into the pipette tips was progressively varied from 5 to 25 µL, and the resulting length of the fibers could be changed from 8 ± 1 to 49 ± 5 mm (**Figures** **2**a and S8, Supporting Information). The length of the prototissue fiber was found to increase linearly with the volume of the concentrated vesicle phase (coefficient of determination: 0.98). To control the diameter of the prototissue fibers, the inner diameter of the pipette tip was instead systematically changed from 370 to 910 µm. Using this strategy, the diameter of the prototissue fibers could be progressively varied from a minimum of 444 ± 7 µm to a maximum of 1240 ± 60 µm (Figures 2b and S9, Supporting Information). The diameter of the fibers was also found to increase linearly with the inner diameter of the tips (coefficient of determination: 0.99). However, through this set of systematic experiments we noticed that the width of the obtained fibers was in average ≈25% larger than the inner diameter of the pipette tip because the prototissues tended to swell slightly when extruded from the pipette tip.

Overall, these results show that our new method enables the controlled assembly of a very high number of vesicle units into robust and freestanding prototissue fibers with controlled lengths and diameters. Our methodology is simple, effective, and highly reproducible. Significantly, since the prototissue fibers could be extruded using a pipette, it was also possible to achieve a spatial control over the assembly of the fibers. Figure 2c,d shows multiple prototissue fibers extruded and spatially organized to display the “BANNO GROUP” text. From a general perspective, these experimental results show that there could be the potential for utilizing our new prototissue fabrication methodology with 3D printing technologies to achieve an even higher spatial resolution and assemble prototissue fibers into complex 3D objects.

### 2.2 Fabrication of Multimodular Fiber Assemblies

**Figure 3:**
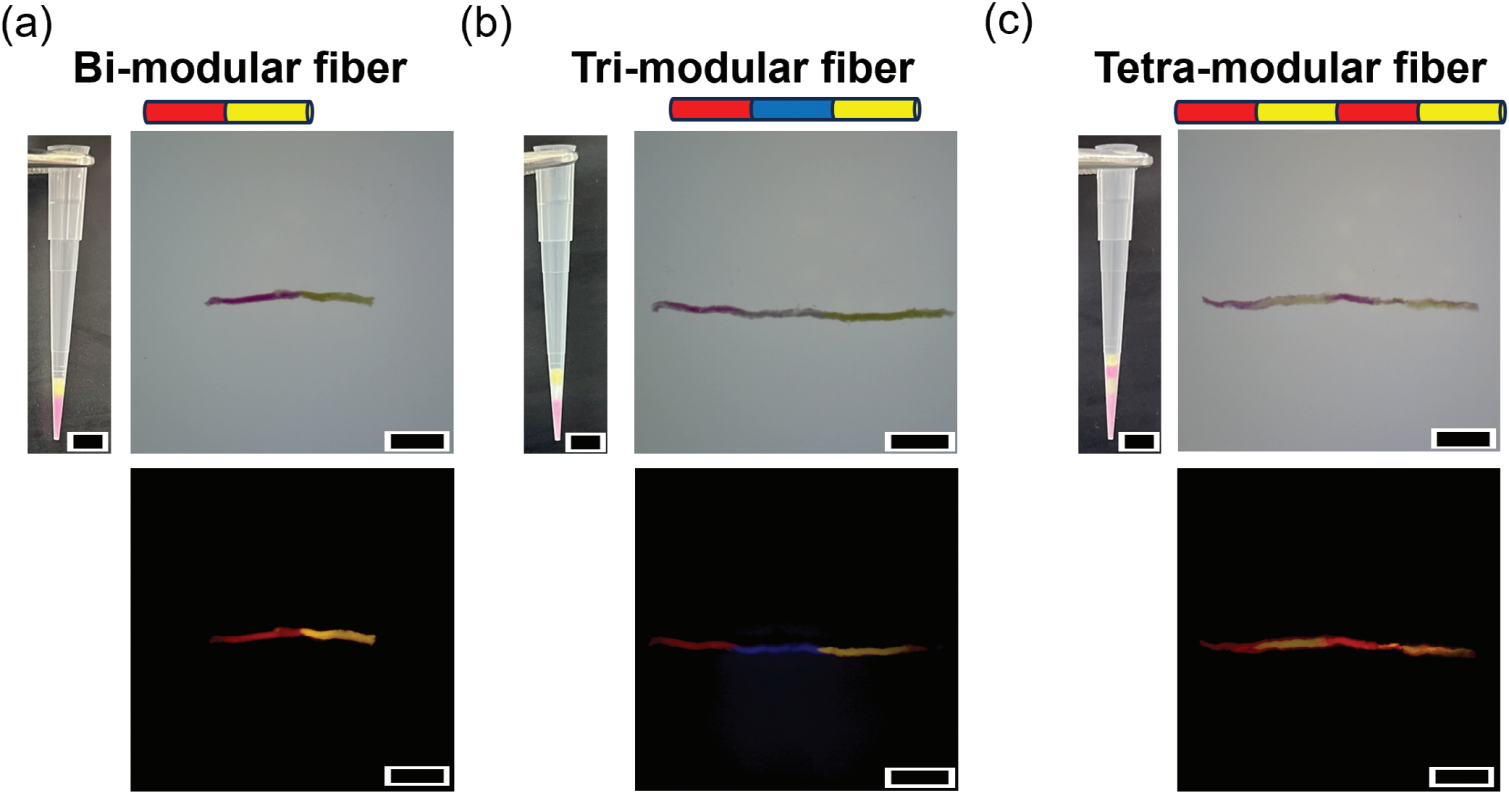
Fabrication of multimodular prototissue fibers. a) A bi‐modular fiber where a Texas Red‐tagged (red fluorescence) fiber and an NBD‐tagged (yellow fluorescence) fiber were connected. b) A tri‐modular fiber where a Texas Red‐tagged (red fluorescence) fiber, a Marina Blue‐tagged (blue fluorescence) fiber, and an NBD‐tagged (yellow fluorescence) fiber were connected. c) A tetra‐modular fiber where Texas Red‐tagged (red fluorescence) fibers and NBD‐tagged (yellow fluorescence) fibers were connected alternately. The excitation and emission wavelength are *λ*_ex_ = 450 nm, *λ*_em_ > 530 nm for Texas Red and NBD; *λ*_ex_ = 365 nm, *λ*_em_ > 420 nm for Marina Blue. Scale bar: 5 mm.

Modularity is ubiquitous in living tissues. The development of prototissue fabrication techniques that allow for a precise spatial segregation of different protocell phenotypes with specific properties and functions is key for the development of more advanced tissue‐like materials, and still remains a technological challenge. Next, we therefore showed that our vesicle extrusion technique allows for the sequential attachment of different fibers composed of different types of protocells. We started by sequentially loading and centrifuging inside the same pipette tip populations of differently tagged vesicles (**Figures** **3** and S10, Supporting Information). The highly packed, layered vesicle populations were then extruded using a mechanical pipette, resulting in modular fiber assemblies. Figure 3a shows a bi‐modular prototissue fiber produced by extruding Texas Red‐tagged (red fluorescence) population of cationic and anionic vesicles, and NBD‐tagged (yellow fluorescence) population of cationic and anionic vesicles. Figure 3b shows instead a tri‐modular prototissue fiber comprising three different vesicle populations, the first population comprised vesicles tagged with Texas Red (red fluorescence), the second population tagged with Marina Blue (blue fluorescence), and the third population tagged with NBD (yellow fluorescence). Another tri‐modular prototissue fiber comprising alternate populations containing Texas Red‐tagged (red fluorescence) vesicles and NBD‐tagged (yellow fluorescence) vesicles was prepared (Figure S11, Supporting Information). Finally, Figure 3c shows a tetra‐modular prototissue fiber composed of alternate populations containing Texas Red‐tagged (red fluorescence) vesicles and NBD‐tagged (yellow fluorescence) vesicles. Importantly, in the prototissue fibers produced, all modules remained well localized, and the differently tagged protocell populations did not mix over time.

**Figure 4:**
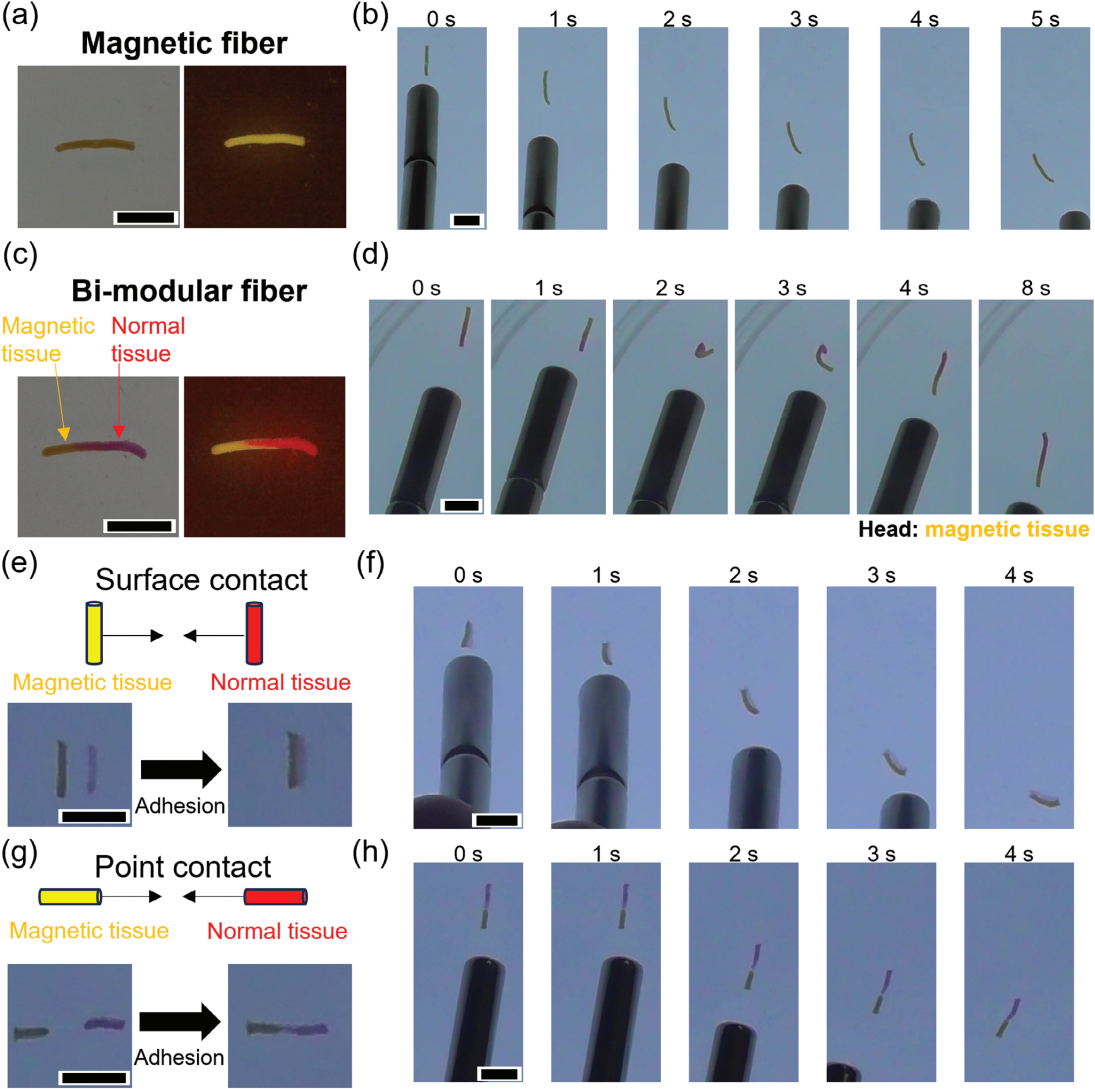
Magnetic manipulation of prototissue fibers. a) Bright‐field and fluorescent images of a magnetic prototissue fiber composed of NBD‐tagged vesicles enclosing magnetic nanoparticles. *λ*_ex_ = 450 nm, *λ*_em_ > 530 nm. Scale bar: 5 mm. b) Magnetic manipulation of the prototissue fiber described in (a). Scale bar: 5 mm. c) A bi‐modular prototissue fiber comprising a driving module capable of magnetotaxis fabricated using NBD‐tagged (yellow fluorescence) vesicles enclosing magnetic nanoparticles, and a cargo module fabricated from Texas Red‐tagged (red fluorescence) vesicles. *λ*_ex_ = 450 nm, *λ*_em_ > 530 nm for Texas Red and NBD; *λ*_ex_ = 365 nm, *λ*_em_ > 420 nm for Marina Blue. Scale bar: 5 mm. d) Magnetic manipulation of the prototissue fiber described in (c), highlighting the possibility of rolling up and unrolling the bi‐modular prototissue fiber by exploiting the magnetotaxis of the driving module (yellow color). Scale bar: 5 mm. e) Connection of an NBD‐tagged magnetic fiber (yellow color, left) to a Texas Red‐tagged normal fiber (red color, right) by their length. Scale bar: 5 mm. f) Magnetic manipulation of the bi‐modular fiber resulting from the attachment of the NBD‐tagged magnetic fiber (yellow color, left) to the Texas Red‐tagged normal fiber (red color, right). Scale bar: 5 mm. g) Connection of an NBD‐tagged magnetic tissue (yellow color, left) and a Texas Red‐tagged normal tissue (red color, right) by their extremities. Scale bar: 5 mm. h) Magnetic manipulation of the bi‐modular fiber resulting from the attachment of the NBD‐tagged magnetic fiber (yellow color, bottom) to the Texas Red‐tagged normal fiber (red color, top). Scale bar: 5 mm.

Next, we explored the possibility of increasing the chemical complexity of the prototissue modules. We thus started by generating magnetic protocells and used them to fabricate a prototissue fiber capable of magnetotaxis. Magnetic nanoparticles were incorporated in cationic and anionic NBD‐tagged vesicles using thin‐film hydration methods (Section S1.4, Supporting Information). Subsequently, the magnetic cationic and anionic vesicles were mixed and concentrated using centrifugation, loaded in a pipette tip, and extruded to generate a prototissue fiber (**Figure** **4**a). Notably, the fiber could move in response to a locally applied magnetic field (Figure 4b and Video S2, Supporting Information), which could also be used to move the fiber through a U‐shaped path (Figure S12 and Video S3, Supporting Information). During magnetotaxis, the fiber remained stable and retained its length and diameter.

Subsequently, we explored the possibility of fabricating modular prototissue fibers capable of magnetotaxis. This could be accomplished through two different strategies. The first strategy involved the possibility of directly extruding a bi‐modular prototissue comprising a driving module for magnetotaxis and a cargo module. The second strategy instead relied on generating two separated modules and joining them by exploiting the interfacial reactivity of the protocell membranes that were capable of salt bridge adhesions. Figure 4c shows a bi‐modular prototissue fiber comprising NBD‐tagged magnetic nanoparticle‐containing vesicles and Texas Red‐tagged vesicles without magnetic nanoparticles that were directly extruded from a pipette tip. The magnetic module of the fiber could be used to drive the entire prototissue fiber via an applied magnetic field. The fiber remained intact during magnetotaxis and could also perform complex twists, rolling ups, and unrolls without breaking (Figure 4d and Video S4, Supporting Information).

Importantly, the same type of modular prototissue fiber could be assembled by joining the driving module for magnetotaxis and the cargo module using salt bridge adhesions (second strategy). Figure 4e shows that the two fiber modules can be joined by their length, and then the entire prototissue can be moved by applying a magnetic field (Figure 4f and Video S5, Supporting Information). Figure 4g shows instead that the two fiber modules can be joined by their tips and then the resulting bi‐modular fiber can undergo magnetotaxis (Figure 4h and Video S6, Supporting Information). In both cases, salt bridges generated prompt and robust adhesions between the two prototissue fibers that could withstand the dragging pull exerted by the driving module.

Overall, these results showcase the versatility of our technique for generation of modular prototissue fibers, and highlight the potential of our new biomimetic materials for applications not only in bottom‐up synthetic biology but also in soft robotics and biomedical engineering.

### 2.3 Combination of Input and Output Modules to Induce Signal Transduction

In the last part of this work, we built on the breakthroughs described above and further advanced the biochemical complexity of the prototissue fibers. We started by fabricating vesicles containing glucose oxidase (GOx) or horseradish peroxidase (HRP). In general, to obtain the enzyme‐containing vesicles, aqueous solutions of the enzymes were used during the thin‐film hydration methods (Section S1.4, Supporting Information).

**Figure 5:**
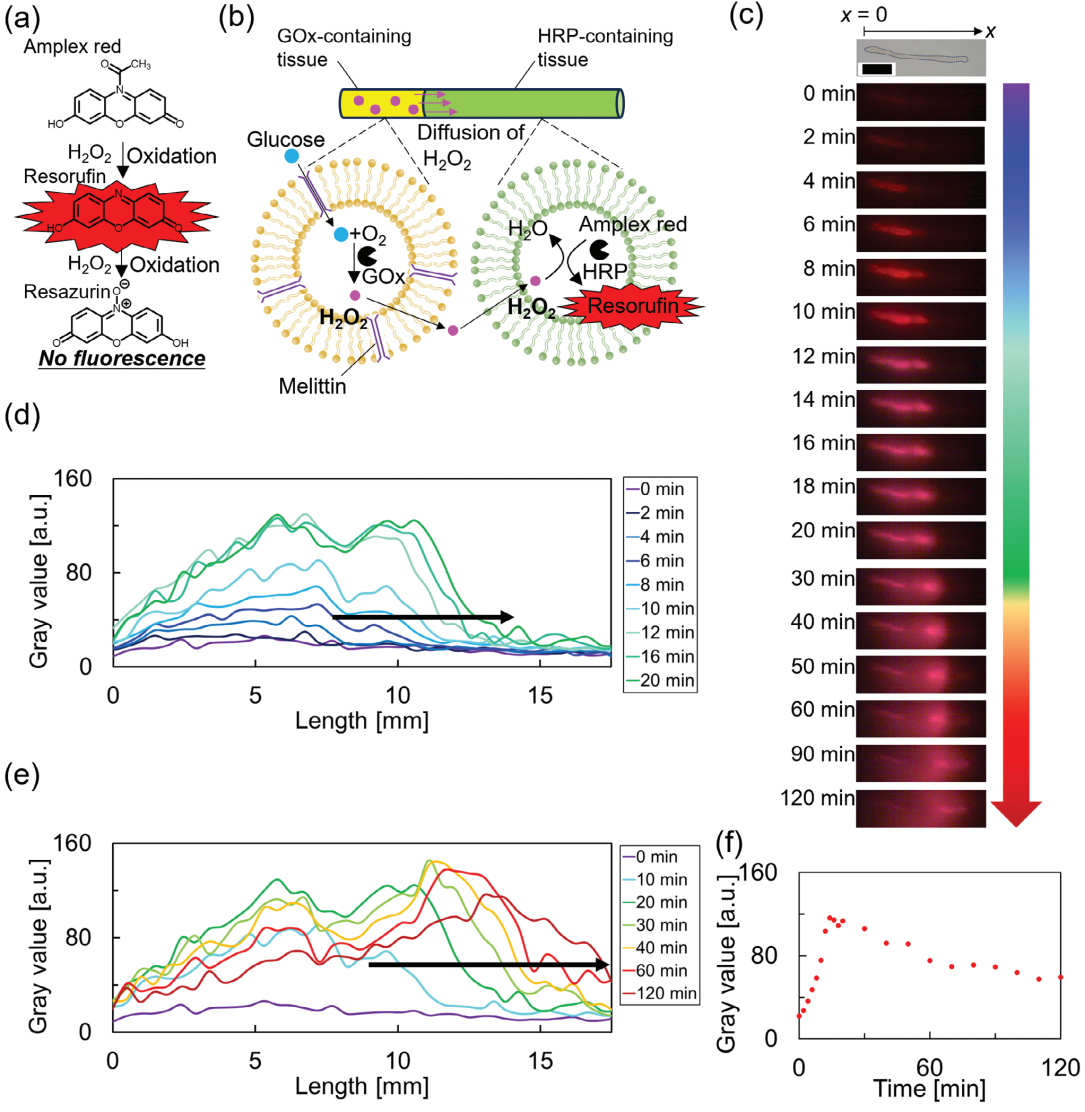
Signal transduction through prototissue fibers connecting input and output modules. a) Mechanism of transient red fluorescence as a consequence of the oxidation of Amplex Red to Resorufin, followed by the oxidation of Resorufin to Resazurin. b) Scheme illustrating the mechanism of the enzyme cascade reaction hosted within the protocells that compose the materials. c) Time‐lapse acquired using digital microscopy upon addition of 100 µL of a solution of glucose (100 × 10^−3^ m) to the prototissue fiber described in (b).* λ*_ex_ = 530 nm, *λ*_em_ > 570 nm. Scale bar: 5 mm. d) Changes in the gray value of (c) obtained by image analysis during 0–20 min. e) Changes in the gray value of (c) obtained by image analysis during 0–120 min. The front of the red fluorescence gradually moved from left to right. f) Time‐dependent changes in the gray value of time‐lapse in (c) obtained by image analysis with ImageJ at the length of 5 mm, where the transient red fluorescence was observed.

Subsequently, we tested the functionality of individual prototissue fibers formed from GOx‐containing vesicles or HRP‐containing vesicles. For this, a fiber made from GOx‐ and melittin‐containing vesicles was placed in an aqueous solution of HRP (≈0.4 U mL^−1^) and Amplex Red (≈0.1 × 10^−3^ m). After adding 100 µL of an aqueous solution of glucose (100 × 10^−3^ m), red fluorescence gradually generated in the bulk aqueous solution (Figure S13, Supporting Information). This indicated permeation of glucose from the bulk aqueous solution into the vesicle through pores made by melittin, with subsequent production and diffusion of H_2_O_2_ from the lumen of the vesicles into the bulk aqueous solution. In the bulk aqueous solution, the dissolved HRP could use H_2_O_2_ to catalyze the oxidation of Amplex Red into red fluorescent Resorufin first and subsequently into nonfluorescent Resazurin (**Figure** **5**a). Similarly, a prototissue fiber made from HRP‐ and Amplex Red‐containing vesicles was placed in an aqueous solution, and 100 µL of an aqueous solution H_2_O_2_ (1 m) was injected on the left end side of the fiber. Red fluorescence generated from the left end of the fiber and gradually moved toward the other end following the diffusion of H_2_O_2_. This indicated permeation of H_2_O_2_ from the bulk aqueous solution into the vesicles that compose the prototissue fiber, with subsequent HRP‐catalyzed oxidation of Amplex Red into Resorufin inside the vesicle lumen (Figure S14 and Video S7, Supporting Information).

Following on these promising results, next, we exploited salt bridge adhesions to assemble a prototissue fiber comprising an input and an output module (Figure 5b). For the input module, we intended a module capable of generating a diffusible chemical signal, whereas for the output module, we intended a module capable of sensing the signal from the input module and generating an output fluorescence signal in response. Specifically, a GOx‐containing fiber was used as the input module because, in the presence of glucose, it is capable of producing the signal H_2_O_2_. An HRP‐containing fiber was used instead as the output module since it is capable of sensing the H_2_O_2_ produced by the input module and catalyzing the oxidation of Amplex Red into Resorufin with consequent red fluorescence. The GOx/HRP bi‐modular prototissue fiber was then placed in an aqueous solution, followed by addition of 100 µL of an aqueous solution of glucose (100 × 10^−3^ m) at the left side of the fiber (Figure S15, Supporting Information). A red fluorescent signal readily generated from the left end side of the HRP containing the output module, which was connected to the GOx containing the input module. Figure 5c–f, Figure S16 and Video S8 (Supporting Information) show that the optical signal progressively moved through the prototissue fiber, highlighting a reaction diffusion front due to a localized formation of the signaling molecule H_2_O_2_ in the input module.

From a general perspective, these results showed that our methodology can be utilized to generate, for the first time, freestanding and reconfigurable prototissue fibers of tightly interconnected protocells with a programmable endogenous reactivity for potential applications in bioengineering and flow chemistry.

## 3 Conclusion

In conclusion, we have reported a novel technique for the extrusion of robust and freestanding prototissue fibers composed of multiple vesicles directly interconnected via salt bridges. Our technique allows for the extrusion of prototissue fibers of controlled lengths, diameters, and shapes, and for the fabrication of modular fibers with transversal sections that are constituted of protocell populations of different phenotypes and specialized functions. The utility and versatility of our technique was then showcased by developing magnetic fiber modules that allowed the fiber to be manipulated using magnetic fields. The magnetic manipulation and the adhesive properties of the fibers could then be synergistically exploited to magnetically drive the attachment of different modules either tip‐to‐tip or side by side. Finally, we showcased the possibility of engineering the fibers with specialized modules for guiding diffusible chemical signals through the fiber itself. This opens up a way to the engineering of reaction diffusion fronts that can propagate directly within tissue‐like materials, while thus far it has only been demonstrated that they can propagate through the bulk aqueous medium using spatially separated periodic arrays of individual protocells or prototissues.^[^ ^16^, ^31^ ^]^

Since our technique for the fabrication of prototissue fibers is based on the extrusion of prototissues through a pipette tip, this should be compatible with applications in 3D printing. We are already exploring this possibility. Thus far only the works of H. Bayley et al. demonstrated the possibility of 3D printing prototissues.^[^ ^17^ ^]^ This would open up the possibility to 3D print prototissues that could be conjugated to failing living tissues, applied to the skin for the release of drugs, or that could be used to fill congenital fistulas, for example.^[^ ^4^, ^32^ ^]^ In fact, the prototissue fibers extruded in this work were made from POPC vesicles. Since liposomes comprising phospholipids such as POPC are known to be biocompatible,^[^ ^33^ ^]^ the fibers are also expected to be biocompatible.

Our prototissue fibers could also find applications as soft robots because they are composed of multiple vesicles with softness similar to bio‐organisms and can carry out mechanical and chemical tasks.^[^ ^4^, ^34^ ^]^ For example, we could build actuators^[^ ^35^ ^]^ or swimmers^[^ ^36^ ^]^ for soft robotics by combining movement modules made of magnetic protocells or of protocells capable of chemical propulsion to manipulation modules that could bend or roll‐up with external physical (light or temperature) or chemical stimuli (pH, chemical gradients). Furthermore, spatiotemporal signal transduction, in which chemical information was transmitted from one side to the opposite side of the fiber, was achieved by combining sensing and transmission modules. This highlights potential for application of the prototissue fibers as soft robots that can sense external stimuli and transmit the chemical signal to other areas of the same tissue‐like material or to another material to trigger a programmed chemical or mechanical response.

From a general perspective, our results pave the way for development of vesicle‐based prototissue fibers as next‐generation bioinspired materials, addressing an important challenge of prototissue engineering. These new materials will find important applications in soft robotics, 3D bio‐printing, microbioreactor technologies, and flow chemistry.

## Conflict of Interest

The authors declare no conflict of interest.

## Supporting information



Supporting Information

Supplemental Video 1

Supplemental Video 2

Supplemental Video 3

Supplemental Video 4

Supplemental Video 5

Supplemental Video 6

Supplemental Video 7

Supplemental Video 8

## Data Availability

The data that support the findings of this study are available in the supplementary material of this article.
