# Supplementary material for: Programmed Fabrication of Vesicle‐Based Prototissue Fibers with Modular Functionalities
Source: Adv Sci (Weinh). 2025 Feb 10;12(16):2409066. doi: 10.1002/advs.202409066 (PMC12021080; doi:10.1002/advs.202409066)
Supplement: Supplementary file 1 — Supporting Information [file ADVS-12-2409066-s003.docx]

Supporting Information

Programmed Fabrication of Vesicle-Based Prototissue Fibers with Modular Functionalities

Tomoya Kojima^1^, Kouichi Asakura^1^, Pierangelo Gobbo*^2,3^, Taisuke Banno*^1^

^1^Department of Applied Chemistry, Keio University, 3-14-1 Hiyoshi, Kohoku-ku, Yokohama, Kanagawa, 223-8522, Japan

^2^Department of Chemical and Pharmaceutical Sciences, University of Trieste, Via L. Giorgieri 1, 34127, Trieste, Italy

^3^National Interuniversity Consortium of Materials Science and Technology Unit of Trieste, Via G. Giusti 9, 50121, Firenze, Italy

**Table of Contents**

1. Materials and methods............................................................................................................2

2. Supplementary figures............................................................................................................7

3. Description of video clips.....................................................................................................21

1. **Materials and methods**

**1.1. Materials**

Hexadecylamine, heptadecanoic acid, Nile red, hydrogen peroxide solutions, glucose, glucose oxidase (GOx), methanol, acetone, and chloroform were purchased from Kanto Chemical Co., Inc (Tokyo, Japan). 1-amidinopyrazole hydrochloride, sucrose and fructose were purchased from Tokyo Chemical Industry (Tokyo, Japan). Horseradish peroxidase (HRP) was purchased from FUJIFILM Wako Pure Chemical (Osaka, Japan). Marina Blue 1,2-dihexadecanoyl-sn-glycero-3-phosphoethanolamine (Marina Blue-DHPE), *N*-(7-nitrobenz-2-oxa-1,3-diazol-4-yl)-1,2-dihexadecanoyl-*sn*-glycero-3-phosphoethanolamine, triethylammonium salt (NBD-PE) and Texas Red 1,2-dihexadecanoyl-*sn*-glycero-3-phosphoethanolamine, triethylammonium salt (Texas Red-DHPE) were purchased from Thermo Fisher Scientific (Waltham, MA). Rhodamine 6G, magnetic nanoparticles (*φ* = 6.4 ± 3.0 nm), melittin and Amplex red were purchased from Sigma-Aldrich (St. Louis, MO). 1-palmitoyl-2-oleoyl-*sn*-glycero-3-phosphocholine (POPC) was purchased from NOF Corporation (Tokyo, Japan). 1 M HEPES buffer (pH 7.2) was purchased from Dojindo Laboratories (Kumamoto, Japan). They were used without further purification.

**1.2 Instrumentation**

^1^H NMR and ^13^C-NMR were carried out using an ECA-500 Fourier transform spectrometer (JEOL Co., Tokyo, Japan), and the chemical shift was based on the TMS peak as 0 ppm. Mass spectrometry was performed by electrospray ionization (ESI) using LCT-Premier (Waters Co., Milford, MA). The samples were prepared by dissolving in acetonitrile.

Digital microscopy (3R-MSUSB401, 3R Solution Co., Ltd., Fukuoka, Japan) was used to observe prototissue fibers precisely. The wavelengths of excitation and emission in the fluorescence images were in the following. *λ*_ex_ = 365 nm and *λ*_em_ > 420 nm for Marina Blue-DHPE, *λ*_ex_ = 450 nm and *λ*_em_ > 530 nm for NBD-PE and Texas Red-DHPE, *λ*_ex_ = 530 nm and *λ*_em_ > 570 nm for Texas Red-DHPE and Resorufin. LED light (LED-EXTA and EX-365, EX-450, EX-530, Optocode Co., Tokyo, Japan) was used as the excitation light.

Confocal laser scanning microscopy (FV10i-DOC, Olympus, Tokyo, Japan) was used to observe prototissue fibers in detail. The wavelengths of excitation and emission in the fluorescence images were in the following. *λ*_ex_ = 473 nm and *λ*_em_ = 490-540 nm for NBD-PE, *λ*_ex_ = 559 nm and *λ*_em_ = 570-620 nm for Texas Red-DHPE and Resorufin. Two photon excitation microscopy (FVMPE-RS, Olympus, Tokyo, Japan) was also used to observe prototissue fibers in detail. The wavelengths of excitation and emission in the fluorescence images were in the following. *λ*_ex_ = 735 nm and *λ*_em_ = 495-540 nm for NBD-PE, *λ*_ex_ = 735 nm and *λ*_em_ = 575-645 nm for Texas Red-DHPE and Resorufin. Fluorescent images obtained by digital microscopy, confocal laser scanning microscopy, or two photon excitation microscopy were analyzed using ImageJ.

Centrifuge 5427 R (Eppendorf Co., Hamburg, Germany) was used for centrifugation.

**1.3. Synthetic procedures**

*Synthesis of guanidium-containing amphiphile*


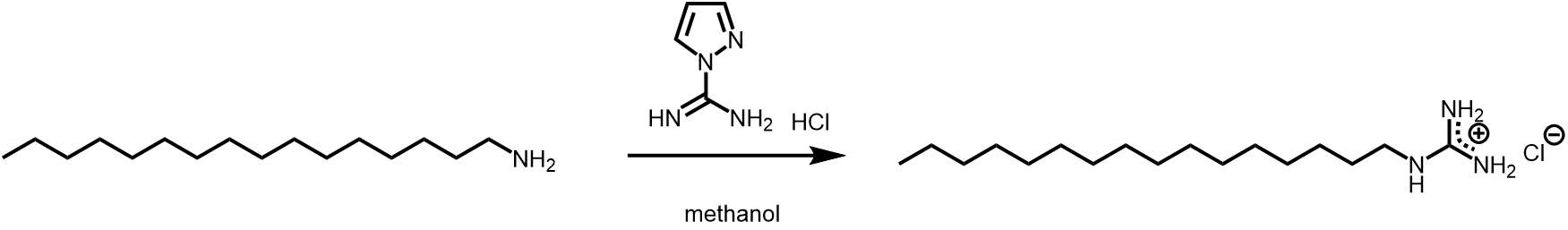


**Scheme S1.** Synthesis of hexadecylguanidium chloride.

*Synthesis of hexadecylguanidium chloride*

Hexadecylamine (270 mg, 1.12 mmol) and 1-amidinopyrazole hydrochloride (164 mg, 1.12 mmol) were dissolved in 5 mL of methanol. The mixture was stirred at 40 ^ο^C under Ar atmosphere for 22 h. After the reaction, the solvent was evaporated, and the crude product was crystallized using 3 mL of acetone to obtain hexadecylguanidium chloride as a white solid (292 mg, 0.910 mmol, 82%).

^1^H-NMR (500 MHz, methanol-*d*_4_) *δ* 3.16 (t, *J* = 7.0 Hz, 2H, -CH_2_-NH-), 1.61-1.55 (m, 2H, -CH_2_-CH_2_-NH-), 1.42-1.23 (m, 28H, CH_3_-(CH_2_)_14_-CH_2_-NH-), 0.90 (t, *J* = 7.0 Hz, 3H, CH_3_-CH_2_-CH_2_-NH-).

^13^C-NMR (126 MHz, DMSO-*d*_6_) *δ* 157.0 (-NH-C-(NH_2_)_2_), 40.6 (-CH_2_-NH-), 31.3 (-CH_2_-CH_2_-NH-), 29.1 (CH_3_-(CH_2_)_5_-(CH_2_)_8_-(CH_2_)_2_-NH-), 29.0 (CH_3_-(CH_2_)_5_-(CH_2_)_8_-(CH_2_)_2_-NH-), 28.7 (CH_3_-(CH_2_)_4_-CH_2_-), 28.7 (CH_3_-(CH_2_)_3_-CH_2_-), 28.5 (CH_3_-(CH_2_)_2_-CH_2_-), 26.1 (CH_3_-CH_2_-CH_2_-), 22.1 (CH_3_-CH_2_-), 13.9 (CH_3_-).

HR-ESI-MS (*m/z*): calcd. for C_17_H_37_ClN_3_ 284.3065 [M-Cl]^+^; found 284.2957 [M-Cl]^+^.

**1.4. Preparation of vesicles using thin-film hydration methods**

Vesicles were prepared using thin-film hydration methods. 2 mL of 10 mM POPC chloroform solution, 10 μL of 100 μM fluorescent lipids or dyes chloroform solution (Marina Blue-DHPE, NBD-PE, Texas Red-DHPE, Rhodamine 6G or Nile red), 160 μL of 5 mM amphiphilic additives chloroform solution (amphiphilic amines, guanidium chloride or carboxylic acids), and 2.83 mL of chloroform were added to a glass vial. The solvent was then removed under reduced pressure for more than 2 h to form a thin film. Then, 10 mL of 10 mM HEPES aqueous solution (pH 7.2) was added to the vial, which was left for 2 h at room temperature to hydrate the film. After 2 h, the vial was agitated using a vortex mixer to form vesicles. The final concentrations were [POPC] = 2 mM, [fluorescent dyes or lipids] = 1 μM, and [amphiphilic additives] = 80 μM.

In order to make vesicles with magnetic nanoparticles, 10 mM HEPES buffer (pH = 7.2) with 0.025 mg/mL magnetic nanoparticles (*φ* = 6.4 ± 3.0 nm) was used to hydrate the films during vesicle formation. Amine- or carboxylic acid-functionalized vesicles containing magnetic nanoparticles were mixed together in a 1:1 ratio to obtain prototissue fibers responsive to magnetic fields. The magnetic manipulation of the fibers was confirmed using a magnet (*φ* 6 mm × 20 mm, 549 mT, AS ONE International, Inc., Santa Clara, CA).

When preparing enzyme-containing cationic vesicles, amphiphilic guanidium chloride was used instead of amphiphilic amines to avoid the influence of the additives on enzymatic reactions. GOx-containing vesicles were prepared using thin-film hydration methods in which 10 mL of HEPES buffer (10 mM, pH = 7.2) containing 2 U/mL GOx, 4 μg/mL melittin, and 200 mM sucrose was used to hydrate the films. HRP-containing vesicles were instead prepared using thin-film hydration methods in which 10 mL of HEPES buffer (10 mM, pH = 7.2) containing 0.2 U/mL HRP, 50 μM Amplex red, and 200 mM sucrose was used to hydrate the films.

**1.5. Preparation of prototissue fibers**

10 mL of dispersions containing cationic vesicles with amphiphilic amines or guanidium chloride and 10 mL of dispersions containing anionic vesicles with amphiphilic carboxylic acids were freshly mixed at the volume ratio of 1:1, and the mixed dispersions were left at least 10 min to promote salt bridges between cationic and anionic vesicles. The vesicle mixture was centrifugated at 12,700 rpm (16,000 *g*) for 10 min to obtain a concentrated vesicle phase. The supernatants were carefully removed. Devices shown in Figure 1b were manually made by using Safe-Lock Tubes 1.5 mL (Eppendorf Co., Hamburg, Germany), polystyrene foam for packaging, and adhesive pads (Hittukimushi, KOKUYO Co., Ltd., Osaka, Japan). The lids of the tubes were removed. An adhesive pad was tightly loaded into the bottom of the tube. A piece of polystyrene foam was loaded to the middle of the tube, and a hole was made at the center of the polystyrene piece. Subsequently, a pipette tip (Pipetman Diamond Tips D200, Gilson Inc., Middleton, WI) was inserted into the hole in the polystyrene piece, and put in tight contact with the adhesive pads present at the bottom of the tube. The concentrated vesicle phase was then loaded into the tip using a mechanical pipette. The devices were centrifugated at 2,000 rpm (400 *g*) for 5 min to obtain highly-packed aggregates of vesicles. Finally, to obtain the prototissue fibers, the tip was carefully removed from the device, and the concentrated vesicle phase was extruded using a mechanical pipette into petri dishes, cuvettes, or glass bottom dishes (D11134H, Matsunami Glass Ind., Ltd., Osaka, Japan) containing an appropriate volume of 10 mM HEPES buffer (pH = 7.2) depending on the type of experiment.

To identify the cationic and anionic vesicles within a fiber, Texas Red-DHPE was loaded within the cationic vesicles, whereas NBD-PE was loaded into the anionic vesicles following the general procedure described in Section 1.4. To characterize the fibers, two photon excitation microscopy or confocal laser scanning microscopy were used.

Volume of concentrated vesicle phase which were loaded into pipette tips was changed from 5 μL to 25 μL when changing the length of the fibers. Whereas, in order to produce prototissue fibers of different diameters, the diameter of the pipette tips which were loaded with the concentrated vesicle phase was changed from 370 μm to 910 μm. The used pipette tips were in the following: Pipetman Diamond Tips D200 (Gilson Inc., Middleton, WI), Pipetman Diamond Tips D1000 (Gilson Inc., Middleton, WI), and Pipette tips epT.I.P.S. Standard 0.5-20 μL 0030000854 (Eppendorf Co., Hamburg, Germany). Our group’s logo “BANNO GROUP” was made with a concentrated vesicle phase by using pipettes as if we wrote letters with a pen.

To prepare multi-modular fibers, the concentrated vesicle phase, which formed the first module, was loaded into pipette tips and centrifugated at 2,000 rpm (400 *g*) for 5 min. After that, another concentrated vesicle phase, which formed a second module, was loaded above the first module and centrifugated at 1,000 rpm (100 *g*) for 5 min. When making tri-modular fibers, an additional concentrated vesicle phase was loaded above the second module and centrifugated at 1,000 rpm (100 *g*) for 5 min. When making tetra-modular fibers, the same procedure was conducted to load the fourth module. The obtained multi-modular vesicles phase was carefully extruded using a mechanical pipette to obtain the desired multi-modular fibers.

**1.6. Signal transduction using enzymatic cascade reactions**

In order to characterize the reactivity of the GOx-containing prototissue fibers, the fibers were immersed in 5 mL of aqueous solutions of HEPES buffer (10 mM, pH = 7.2), sucrose (100 mM), and fructose (100 mM). 10 μL of 200 U/mL HRP, and 50 μL of 10 mM Amplex red were added to the aqueous solutions. 100 μL of 100 mM glucose solutions were added to the overall petri dish, and red fluorescence derived from the oxidation of Amplex Red to Resorufin was observed over time using digital microscopy.

In order to characterize the reactivity of the HRP-containing prototissue fibers, the fibers were instead immersed in 5 mL of aqueous solutions of HEPES buffer (10 mM, pH = 7.2), sucrose (100 mM), and fructose (100 mM). 100 μL of 1 M H_2_O_2_ solutions were added to the left side of the fibers, and red fluorescence derived from the oxidation of Amplex Red to Resorufin was observed over time using digital microscopy.

In order to characterize signal transduction in prototissue fibers comprising an input and output module, GOx- and HRP-containing prototissue fibers were connected based on the multi-modular techniques as shown in Section 1.5. The obtained bi-modular fiber was immersed in 5 mL of aqueous solutions of HEPES buffer (10 mM, pH = 7.2), sucrose (100 mM), and fructose (100 mM). 100 μL of 100 mM glucose solutions were added to the edge of the GOx-containing prototissue fibers, and red fluorescence derived from Resorufin was observed.

1. **Supplementary figures**


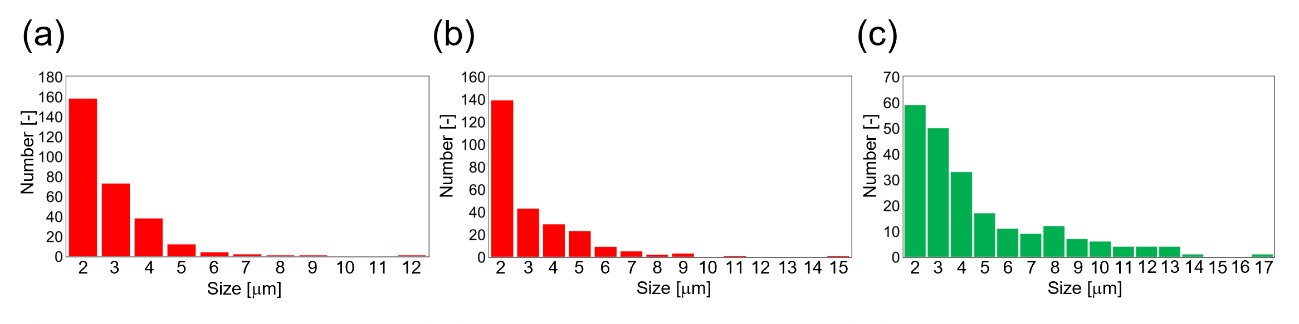


**Figure S1.** Size distribution of vesicles containing (a) amphiphilic amine, (b) amphiphilic guanidium, and (c) amphiphilic carboxylic acid. Conditions: [POPC] = 500 μM, [amphiphilic additive] = 20 μM, [fluorescent lipid] = 1.0 μM.


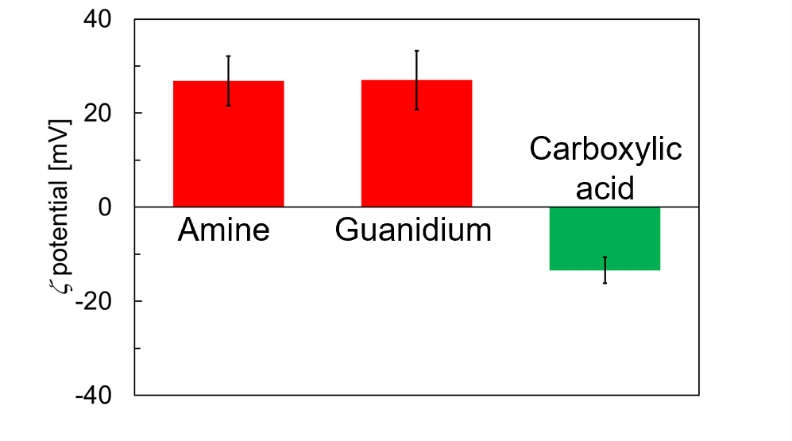


**Figure S2.** *ζ* potential of vesicles containing amphiphilic amine, guanidium, and carboxylic acid. Conditions: [POPC] = 500 μM, [amphiphilic additive] = 20 μM.


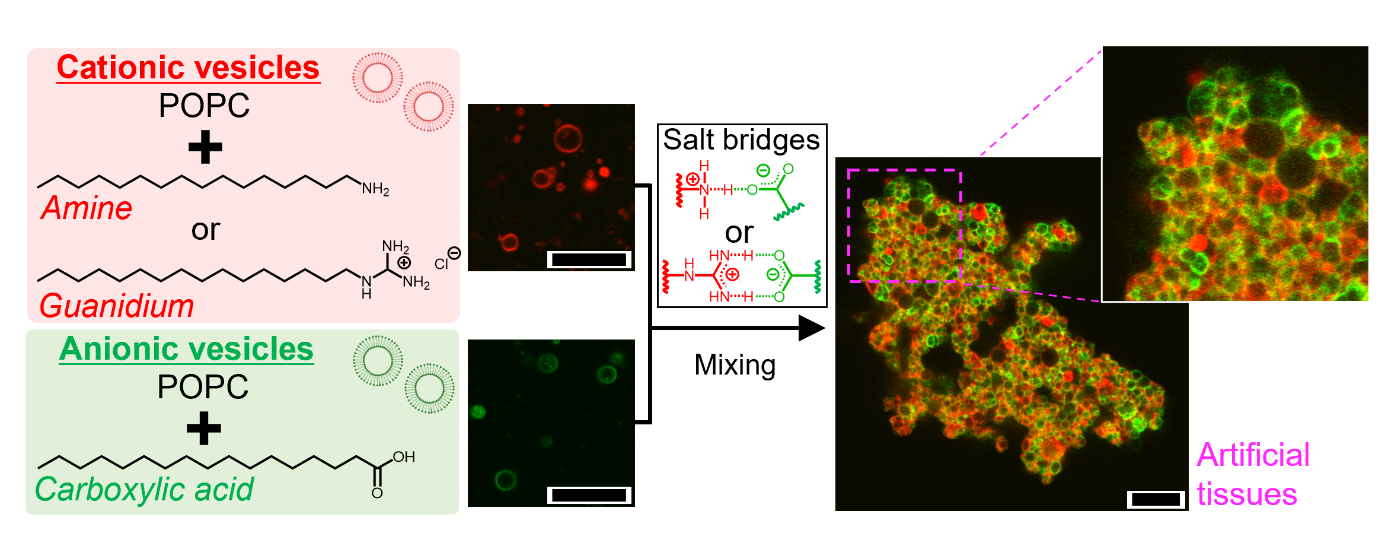


**Figure S3.** Adhesion of cationic and anionic vesicles *via* salt bridges. On the left are reported the schematic structures and the corresponding confocal laser scanning microscopic images of Texas Red-tagged (red fluorescence) cationic vesicles and NBD-tagged (green fluorescence) anionic vesicles. On top of the arrow is a scheme describing the two types of salt bridges that can be generated when mixing cationic and anionic vesicles. On the right of the arrow, confocal laser scanning microscopic images showing a concentrated vesicle phase held together by salt bridges is reported. Scale bar: 200 μm.


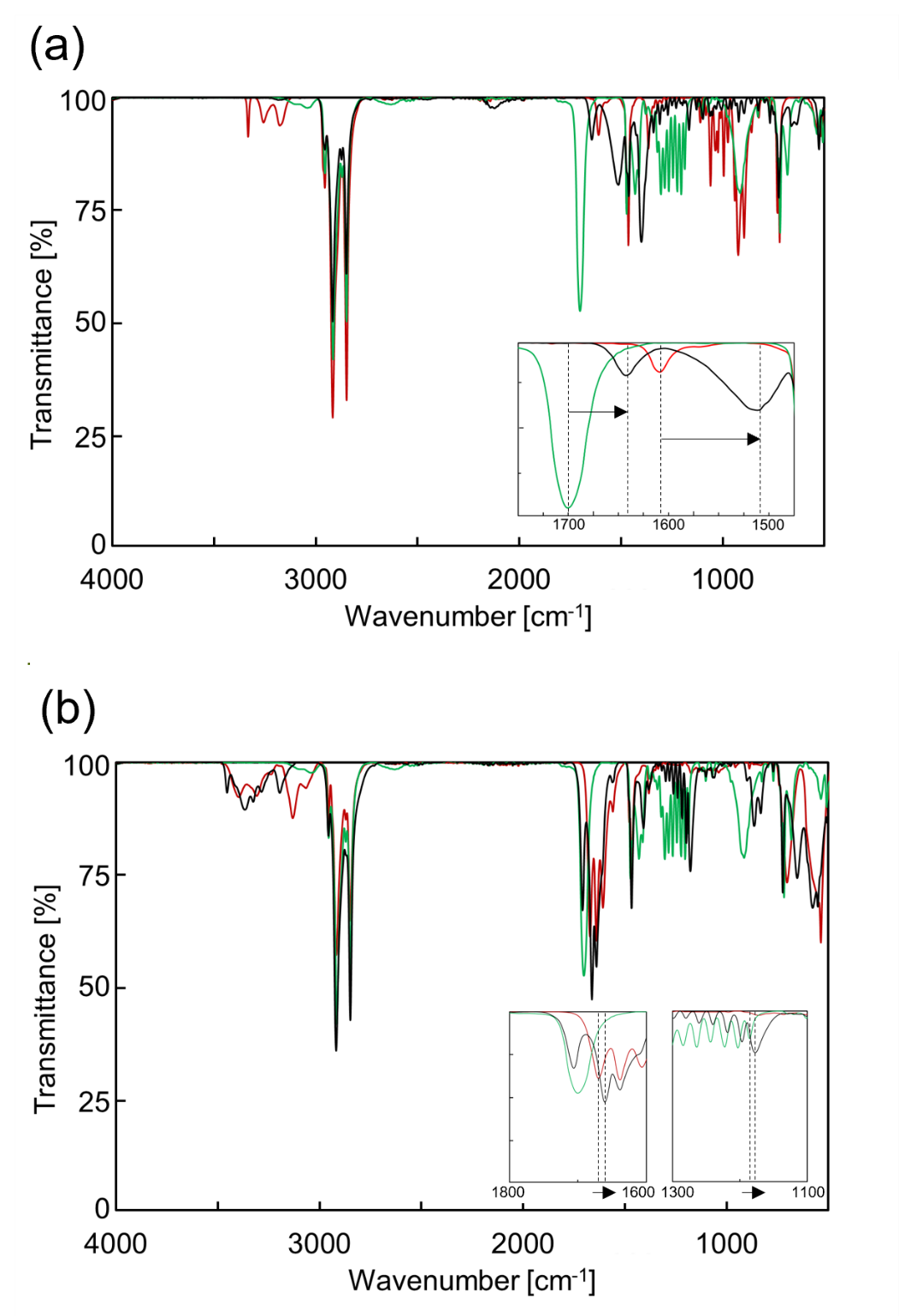


**Figure S4.** IR spectra of amphiphilic additives. (a) IR spectra of amphiphilic amine (red), amphiphilic carboxylic acid (green), and their mixtures (black). The inset shows the shifts for both C═O stretching vibrations and N-H bending vibrations to lower wavenumbers, which indicated the formation of hydrogen bonds. (b) IR spectra of amphiphilic guanidium (red), amphiphilic carboxylic acid (green), and their mixtures (black). The insets show the shifts for both N-H bending vibrations and C-O stretching vibrations to lower wavenumbers, which indicated the formation of hydrogen bonds.


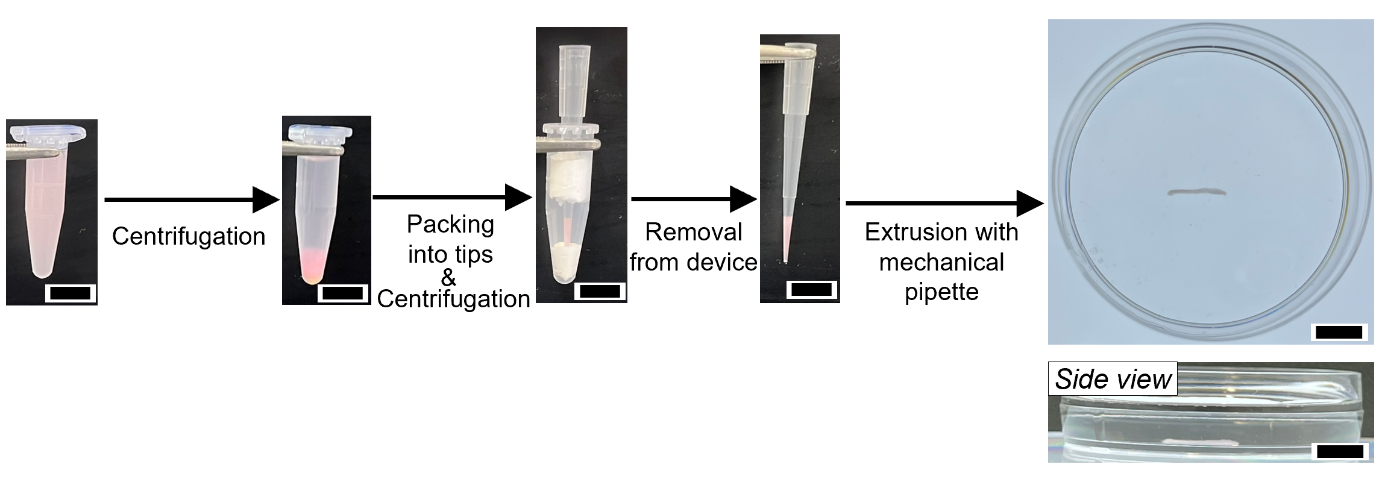


**Figure S5.** Detailed methods to fabricate prototissue fibers comprising amine-functionalized vesicles tagged with Texas Red-DHPE and carboxylic acid-functionalized vesicles tagged with NBD-PE. Scale bar: 1 cm.


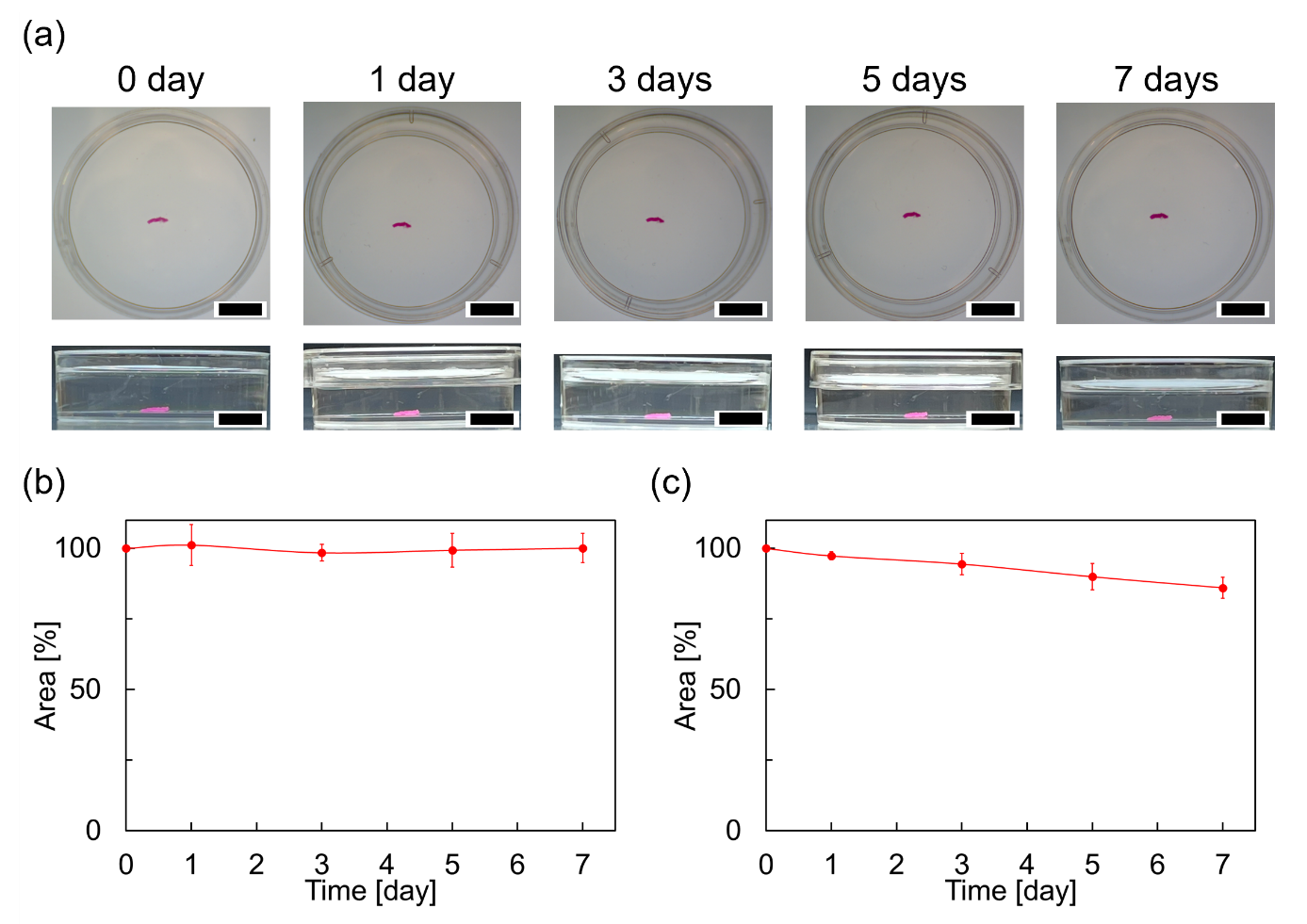


**Figure S6.** Stability of prototissue fibers over time in 10 mM HEPES buffer. (a) Time-lapse showing the stability of a prototissue fiber at room temperature (23-25 °C) over 1 week. The fiber was composed of amine-functionalized cationic vesicles and carboxylic acid-functionalized anionic vesicles tagged with Nile red. Scale bar: 5 mm. (b) Plot showing the time-dependent changes in the area of the prototissue fiber at room temperature (23-25 °C) in (a). The ratio was calculated based on the area at day 0. The absence of changes in the area of the fiber indicates that the fibers did not dissolve and retained their structures over a long time. (c) Plot showing the time-dependent changes in the area of the prototissue fiber at 37 °C. Although the fiber got a little bit smaller over time, it remained stable after 7 days.


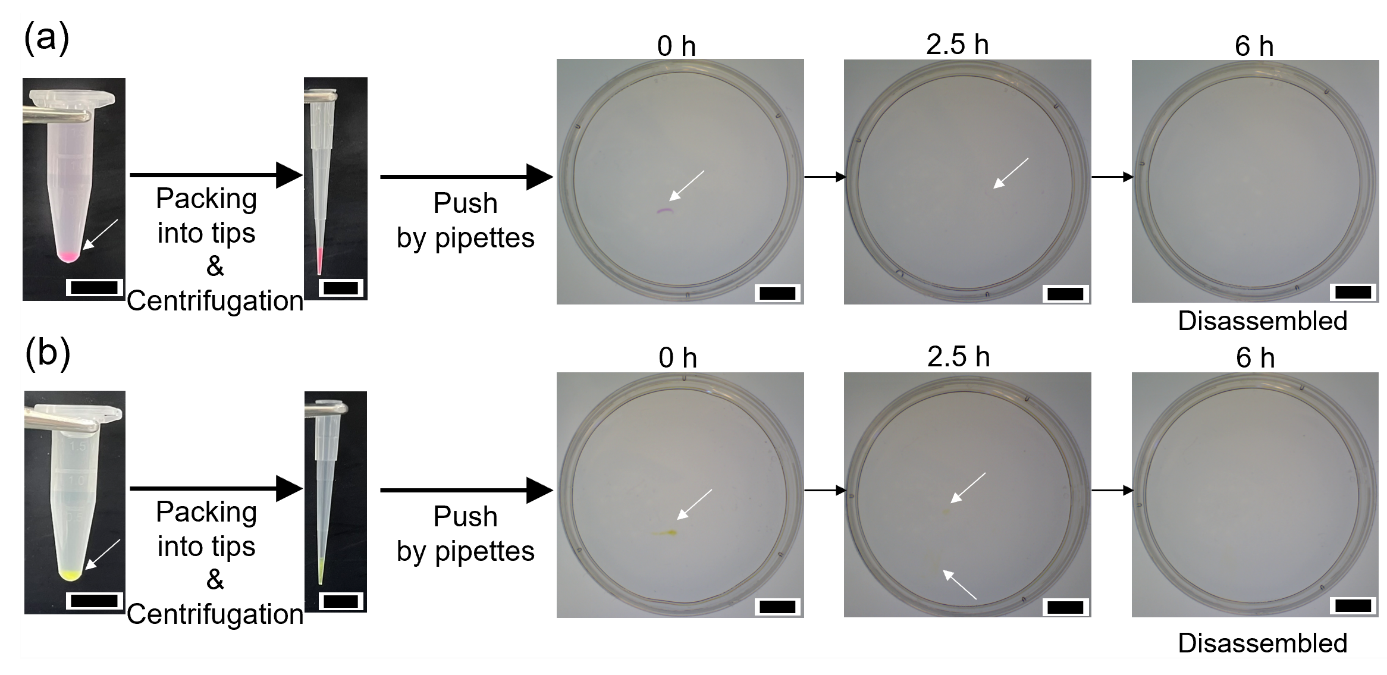


**Figure S7.** Control experiments where we tried to fabricate prototissues fibers using only (a) amine-functionalized cationic vesicles or (b) carboxylic acid-functionalized anionic vesicles. In both control experiments, the fibers readily disassembled. Scale bar: 1 cm.


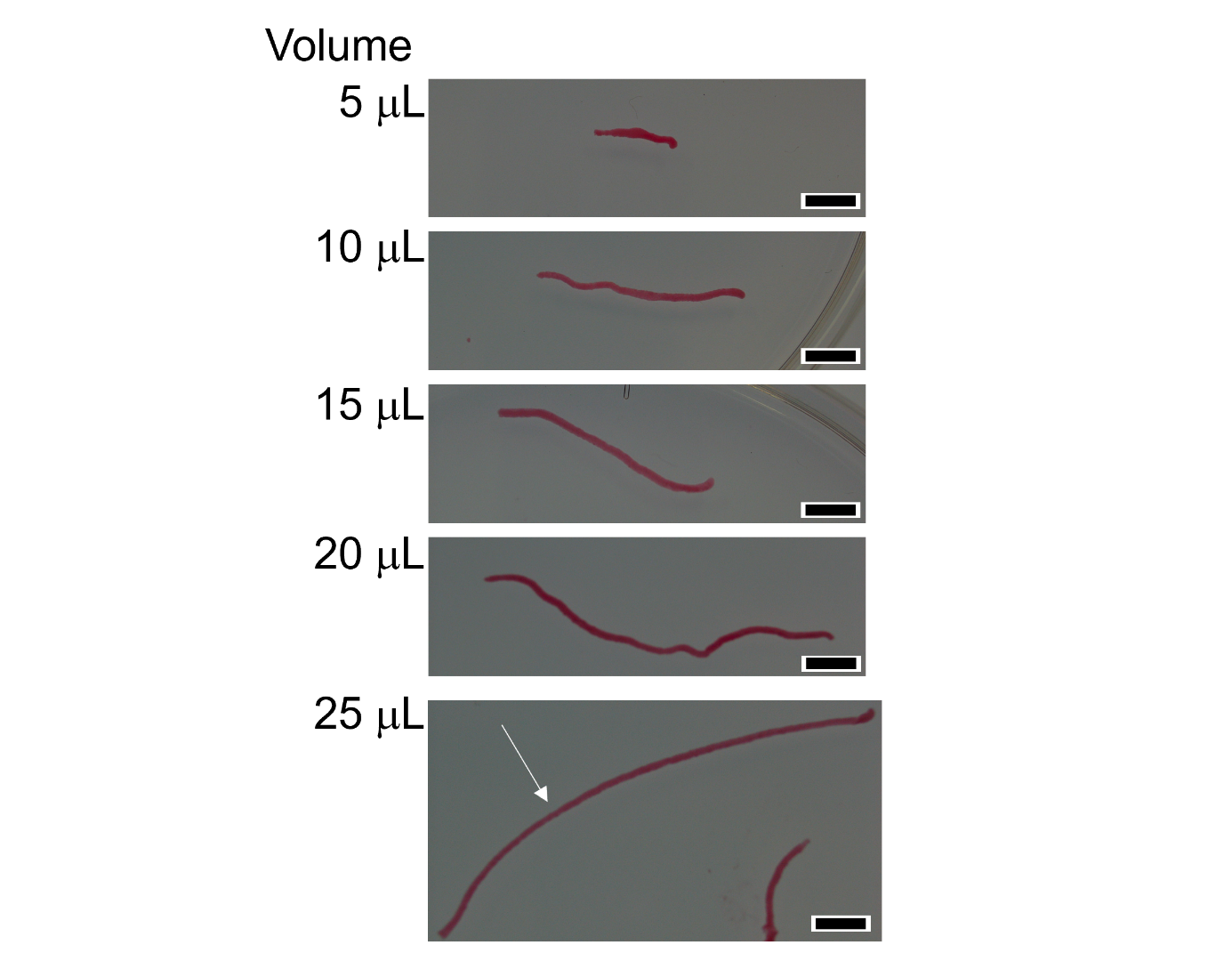


**Figure S8.** Prototissue fibers of different lengths composed of amine-functionalized and carboxylic acid-functionalized vesicles tagged with Nile red. The fibers were produced by systematically increasing the volume of concentrated vesicle phase in the pipette tip. The volumes used are reported on the top left corner of the corresponding image. Scale bar: 5 mm.


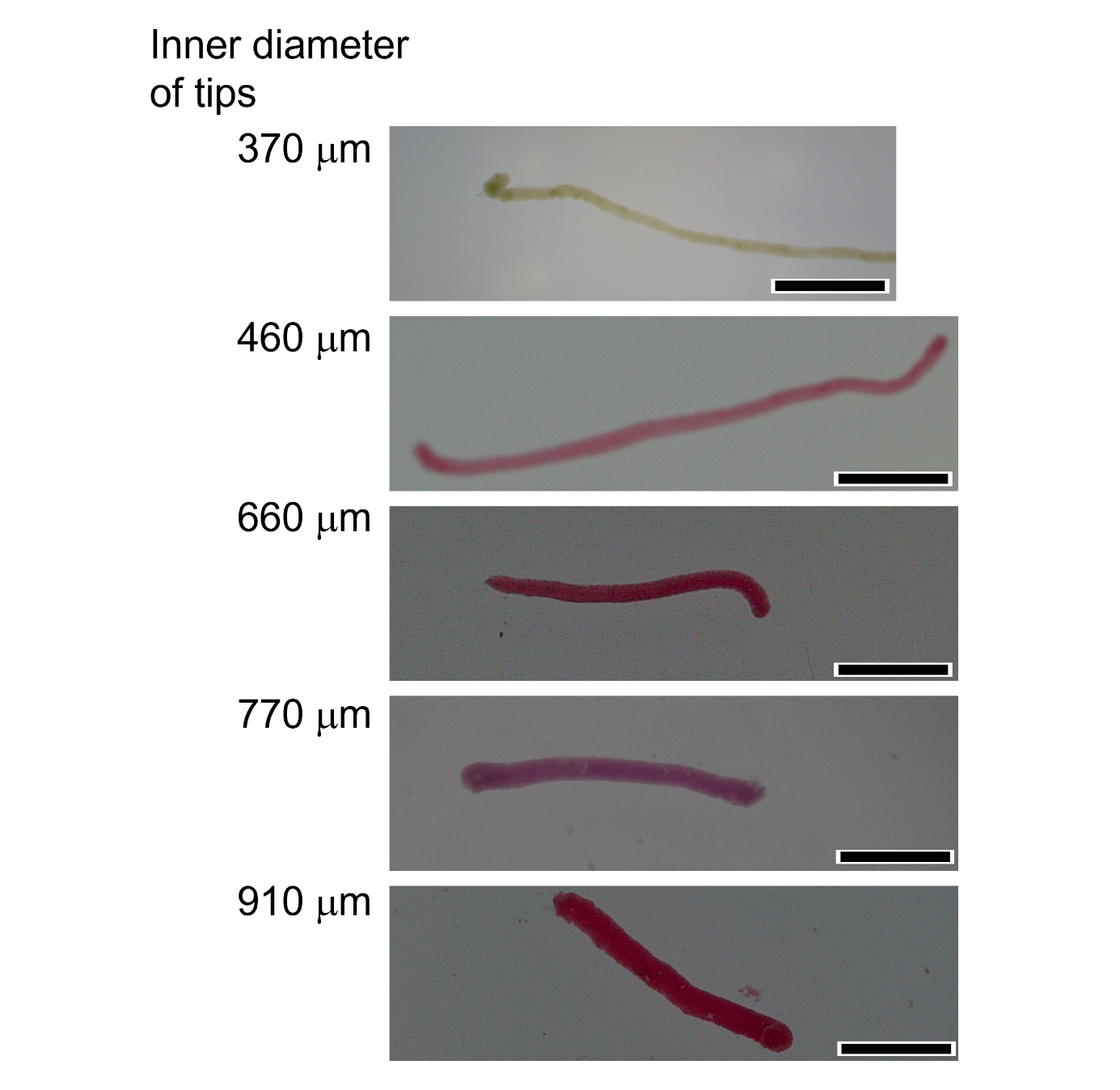


**Figure S9.** Prototissue fibers composed of amine-functionalized and carboxylic acid-functionalized vesicles tagged with NBD-PE (for the 370 μm diameter) or Nile red (for the 460–910 μm dimeter), which were produced with different diameters by changing the inner diameter of pipette tip used for their extrusion. The inner diameter of the tip is reported on top left corner of the corresponding image. Scale bar: 5 mm.


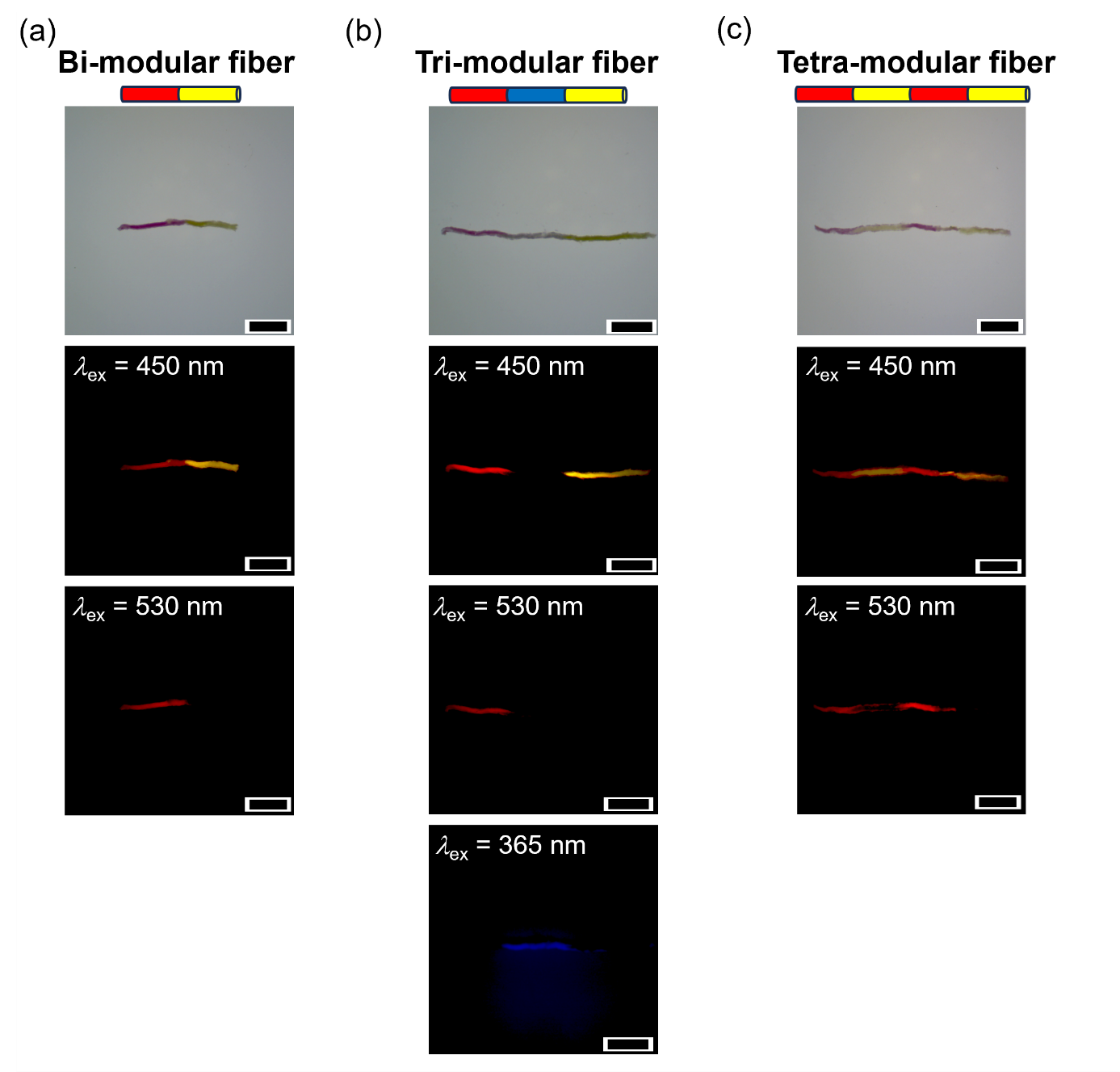


**Figure S10.** Multi-modular prototissue fibers with (a) two, (b) three, and (c) four different modules irradiated with light at the different excitation wavelength, as reported on the top of each image. The red blocks comprise Texas Red-tagged vesicles, the yellow blocks comprise NBD-tagged vesicles, and the blue blocks comprise Marina Blue-tagged vesicles. The wavelength of excitation and emission are as follows: *λ*_ex_ = 365 nm, *λ*_em_ > 420 nm; *λ*_ex_ = 450 nm, *λ*_em_ > 530 nm; *λ*_ex_ = 530 nm, *λ*_em_ > 570 nm. Scale bar: 500 μm.


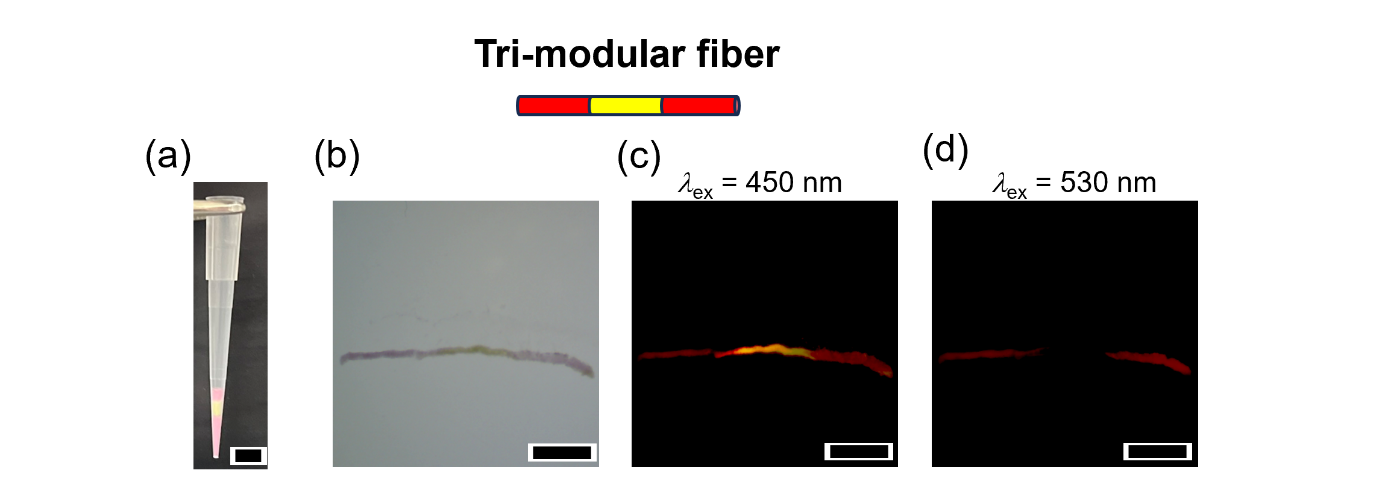


**Figure S11.** Tri-modular prototissue fiber. (a) An image of pipette tips loaded with three modules. (b-d) Bright-field (b) and fluorescent images (c, d) of the tri-modular prototissue fiber using digital microscopy. The two red blocks comprise Texas Red-tagged vesicles, whereas the yellow block comprises NBD-tagged vesicles. The wavelength of excitation and emission are as follows: *λ*_ex_ = 450 nm, *λ*_em_ > 530 nm; *λ*_ex_ = 530 nm, *λ*_em_ > 570 nm. Scale bar: 500 μm.


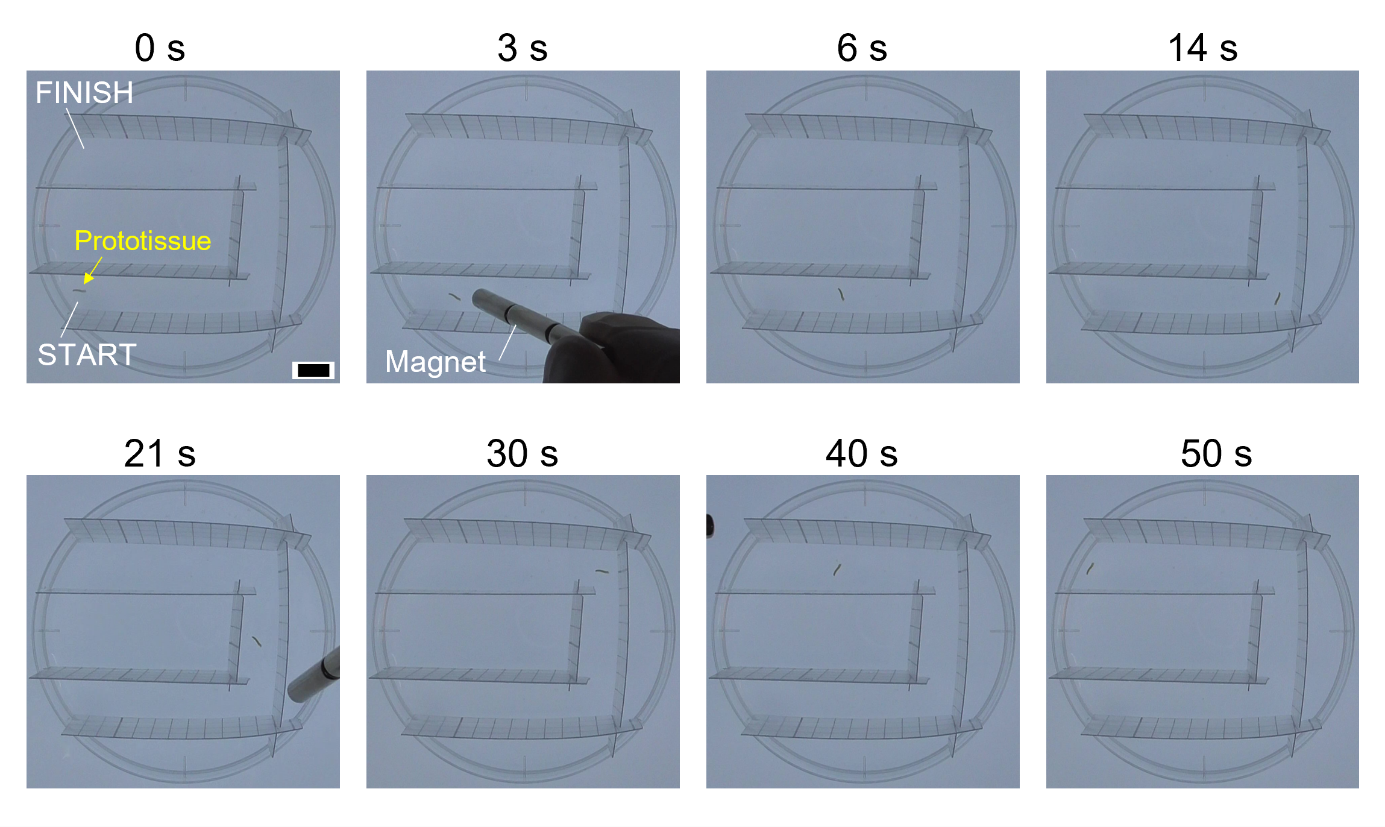


**Figure S12.** Magnetic manipulation of a magnetic prototissue fiber along a U-shaped path. Scale bar: 1 cm.


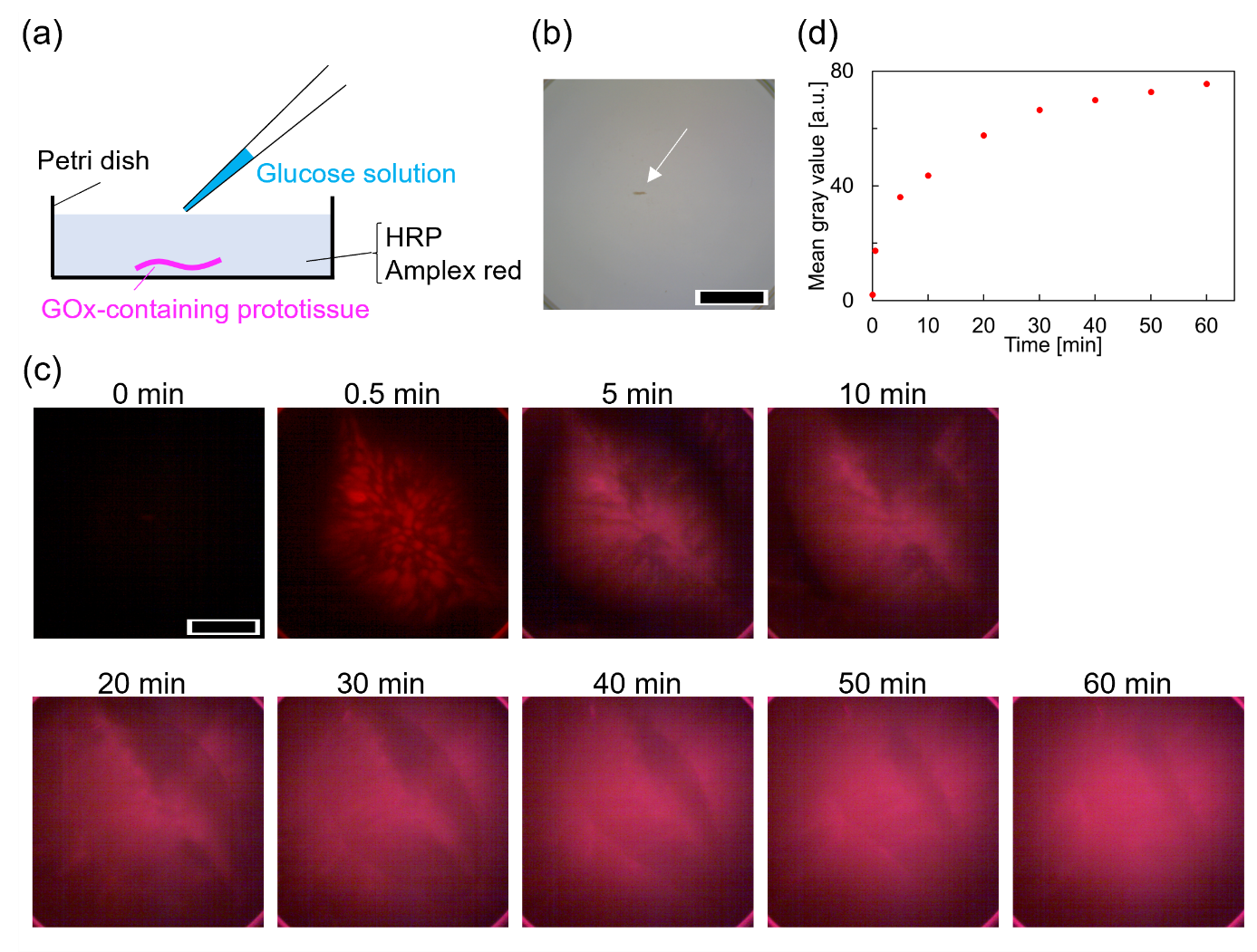


**Figure S13.** Enzymatic reactivity of a GOx-containing prototissue fiber. (a) Schematic illustration of experiments. Glucose solutions (100 mM, 100 μL) were added to a GOx-containing prototissue fiber placed in an aqueous solution (5 mL) containing HRP (*ca.* 0.4 U/mL) and Amplex red (*ca.* 0.1 mM). (b) An image of the GOx-containing prototissue fiber described in (a) taken from the top. Scale bar: 1 cm. (c) Digital microscopy images showing the time-dependent red fluorescence turn-on of the solution surrounding the GOx-containing prototissue fiber described in (a) upon addition of an aqueous glucose solution (100 mM, 100 μL) due to the production of Resorufin. Scale bar: 1 cm. (d) Plot showing the time-dependent changes in the fluorescence intensity of bulk solutions for the experiment described in (c).


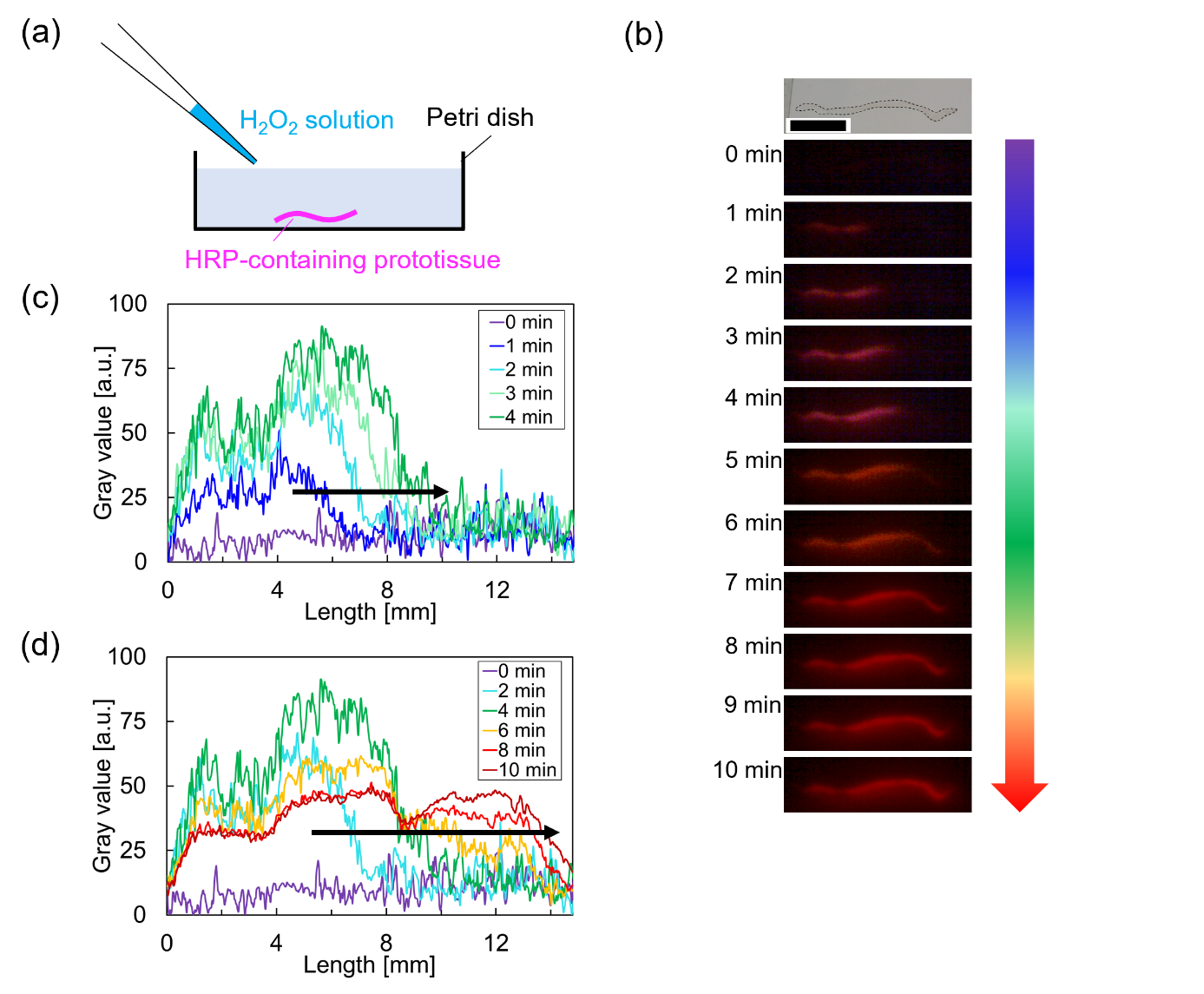


**Figure S14.** Enzymatic reactions of HRP-containing prototissue fibers. (a) Schematic illustration of the experiment. H_2_O_2_ solutions (1 M, 100 μL) were added to the left side of a HRP-containing prototissue fiber (0.2 U/mL) with Amplex Red (50 μM) and placed in an aqueous solution (5 mL). (b) Time-lapse images of the HRP-containing fiber after adding the H_2_O_2_ solution (1 M, 100 μL) at the left side of the fiber. *λ*_ex_ = 530 nm; *λ*_em_ >570 nm to capture the synthesis of Resorufin. Scale bar: 5 mm. (c) Changes in the gray value of Figure 5b obtained by image analysis during 0-4 min. (d) Changes in the gray value of Figure 5b obtained by image analysis during 0-10 min. The front of the red fluorescence gradually moved from left to right.


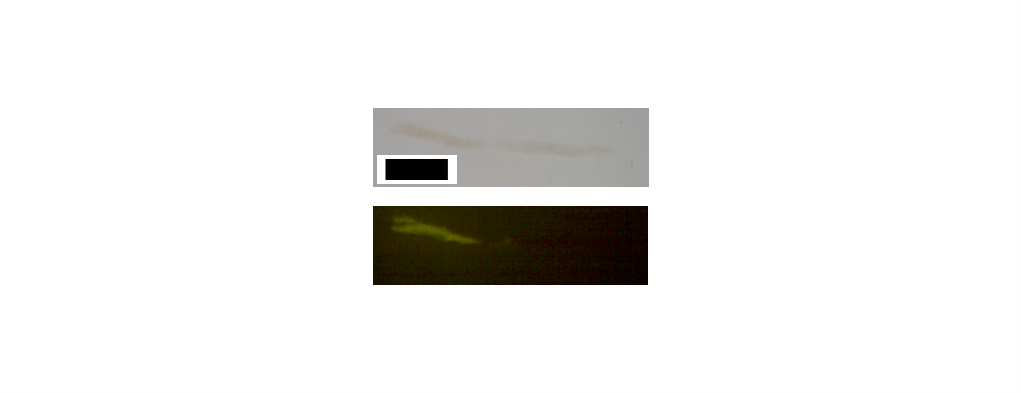


**Figure S15.** Images of a prototissue fiber comprising a module of GOx-containing NBD-PE-tagged vesicles (yellow fluorescence, left), followed by a module of non-tagged HRP-containing vesicles (right). Scale bar: 5 mm.


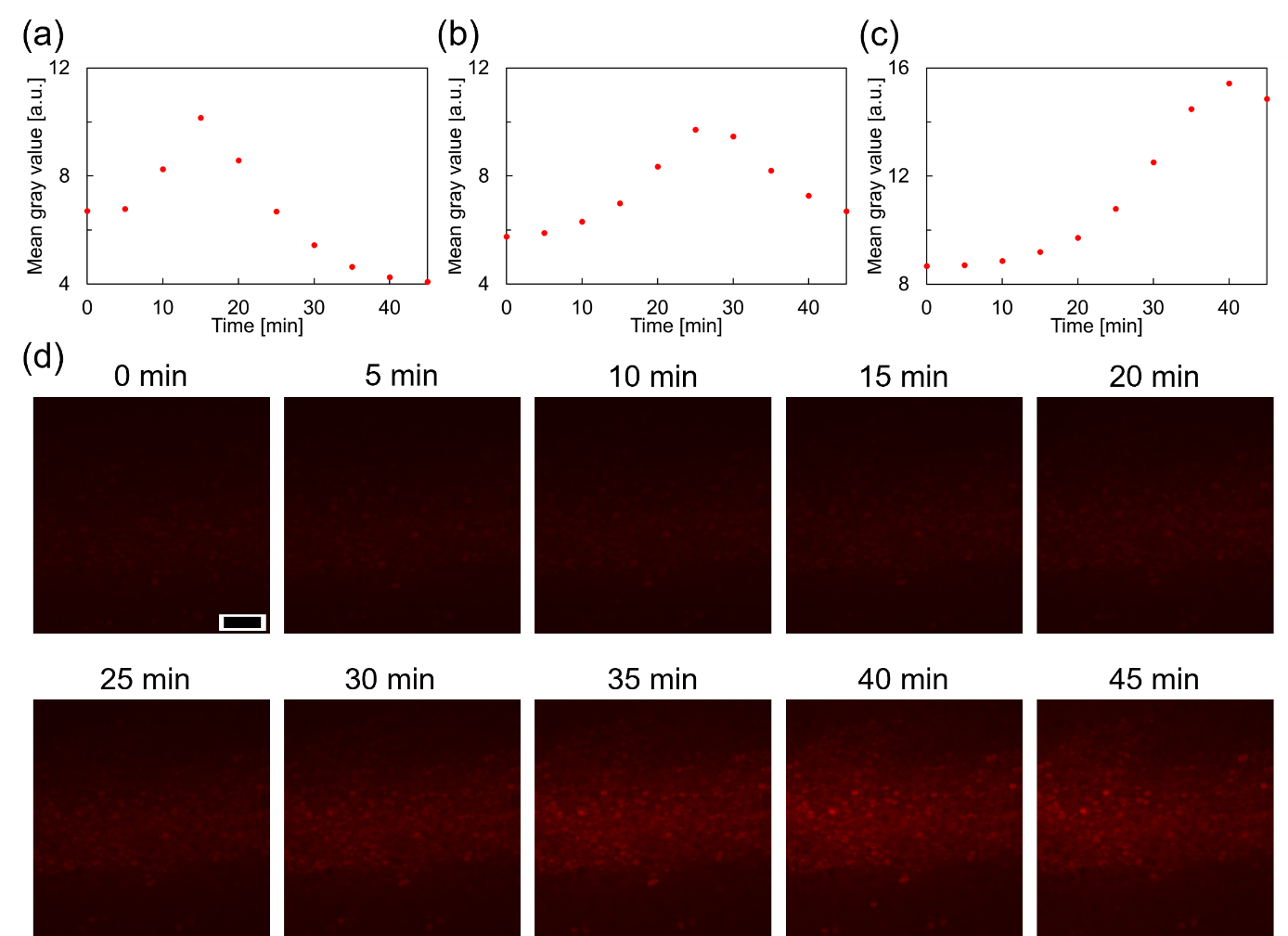


**Figure S16.** (a-c) Time-dependent changes in the mean gray value at confocal laser scanning microscopic images focusing only on an output prototissue fiber module containing HRP. An input prototissue fiber module that contained HRP (0.2 U/mL) and Amplex Red (50 μM), and an output prototissue fiber module with GOx-containing vesicles (2 U/mL) which comprised melittin (4 μg/mL) were connected to obtain bi-modular fibers. Glucose solutions (100 mM, 100 μL) were added to the GOx-containing prototissue fiber placed in aqueous solutions. Three different trials were shown in (a)-(c). (d) Time-dependent confocal laser scanning microscopic images in (c). Scale bar: 200 μm.

**3. Description of video clips**

**Supplementary video 1.** Fabrication of a prototissue fiber ejected by a pipette tip.

**Supplementary video 2.** Manipulation of a magnetic prototissue fiber in response to magnets.

**Supplementary video 3.** Manipulation of a magnetic prototissue fiber along a U-shaped aisle in response to magnets.

**Supplementary video 4.** Magnetic manipulation of a bi-modular fiber where the magnetic tissue (yellow) and the normal tissue (red) were connected.

**Supplementary video 5.** Magnetic manipulation of tissues where the magnetic tissue (yellow) and the normal tissue (red) were adhered at a surface contact.

**Supplementary video 6.** Magnetic manipulation of tissues where the magnetic tissue (yellow) and the normal tissue (red) were adhered at a point contact.

**Supplementary video 7.** Signal transduction of red fluorescence derived from Resorufin using HRP-containing prototissues when H_2_O_2_ solutions were added.

**Supplementary video 8.** Signal transduction of red fluorescence derived from Resorufin using a bi-modular prototissue fiber composed of GOx-containing and HRP-containing prototissues when glucose solutions were added.
